# IFNγ drives neuroinflammation, demyelination, and neurodegeneration in a mouse model of multiple system atrophy

**DOI:** 10.1186/s40478-023-01710-x

**Published:** 2024-01-18

**Authors:** Nicole J. Corbin-Stein, Gabrielle M. Childers, Jhodi M. Webster, Asta Zane, Ya-Ting Yang, Nikhita Mudium, Rajesh Gupta, Fredric P. Manfredsson, Jeffrey H. Kordower, Ashley S. Harms

**Affiliations:** 1 Department of Neurology, Center for Neurodegeneration and Experimental Therapeutics, University of Alabama at Birmingham, 1719 6th Ave South, Birmingham, AL 35294 USA; 2 Department of Translational Neuroscience, Barrow Neurological Institute, Phoenix, AZ USA; 3 ASU-Banner Neurodegenerative Disease Research Center, Biodesign Institute, Arizona State University, Tempe, AZ USA

**Keywords:** Multiple system atrophy, Neuroinflammation, Alpha-synuclein, T cells, Interferon gamma

## Abstract

Multiple system atrophy (MSA) is a rare and fatal synucleinopathy characterized by insoluble alpha-synuclein (α-syn) cytoplasmic inclusions located within oligodendroglia. Neuroinflammation, demyelination, and neurodegeneration are correlated with areas of glia cytoplasmic inclusions (GCI) pathology, however it is not known what specifically drives disease pathogenesis. Recent studies have shown that disease pathologies found in post-mortem tissue from MSA patients can be modeled in rodents via a modified AAV overexpressing α-syn, Olig001-SYN, which has a 95% tropism for oligodendrocytes. In the Olig001-SYN mouse model, CD4+ T cells have been shown to drive neuroinflammation and demyelination, however the mechanism by which this occurs remains unclear. In this study we use genetic and pharmacological approaches in the Olig001-SYN model of MSA to show that the pro-inflammatory cytokine interferon gamma (IFNγ) drives neuroinflammation, demyelination, and neurodegeneration. Furthermore, using an IFNγ reporter mouse, we found that infiltrating CD4+ T cells were the primary producers of IFNγ in response to α-syn overexpression in oligodendrocytes. Results from these studies indicate that IFNγ expression from CD4+ T cells drives α-syn-mediated neuroinflammation, demyelination, and neurodegeneration. These results indicate that targeting IFNγ expression may be a potential disease modifying therapeutic strategy for MSA.

**Supplementary Information:**

The online version contains supplementary material available at 10.1186/s40478-023-01710-x.

## Introduction

Multiple system atrophy (MSA) is a rare, rapidly progressive, and fatal demyelinating synucleinopathy with no known disease modifying therapy [8, 33]. Unlike other synucleinopathies such as Parkinson disease (PD), MSA pathology is characterized by severe autonomic failure and rapidly progressive demyelination and neurodegeneration associated with alpha-synuclein (α-syn) containing glial cytoplasmic inclusions (GCIs) within oligodendrocytes [8, 25, 31]. MSA is divided into two subtypes, MSA-Parkinsonian (MSA-P) and MSA-Cerebellar (MSA-C). MSA-P is the most common in western countries, affecting 80% of patients and neurodegeneration occurs primarily within the striatonigral pathway [8]. MSA-C leads to olivopontocerebellar atrophy [8]. Although the areas of degeneration differ depending on MSA subtype, GCI pathology is present in vulnerable and resistant regions across the neuraxis. GCI pathology is associated with significant neuroinflammation, demyelination, and neurodegeneration [8, 19, 33]. However, the mechanism by which GCI pathology leads to neuroinflammation, demyelination, and neurodegeneration is currently unknown.

Previous studies have shown neuroinflammation as a pathological hallmark of MSA. In MSA post-mortem brains, widespread astrogliosis and microgliosis are present within areas of neurodegeneration and demyelination [8, 19, 33]. The expression of major histocompatibility complex II (MHCII), the equivalent of human leukocyte antigen-DR (HLA-DR), is found on antigen presenting cells (APCs) within the Central Nervous System (CNS) [35]. In post-mortem MSA brains, our previous study showed that α-syn GCI pathology is accompanied by MHCII+ expression and increased infiltration of peripheral T cells (CD4+ , CD8+) [35]. Using a novel modified AAV in which human α-syn is overexpressed in oligodendroglia (Olig001-SYN) [21, 26] we observed significant neuroinflammation and demyelination. Thus, effectively modeling MSA in rodents and non-human primates [22]. Using this Olig001-SYN model of MSA, we also demonstrated significant MHCII expression on CNS resident microglia and infiltrating monocytes and macrophages, along with infiltration of CD4+ and CD8+ T cells, similar to that observed in post-mortem brains [35]. Using mice that are genetically deficient in CD4+ T cells, we found that Olig001-SYN induced-MHCII expression, infiltration of peripheral immune cells, and demyelination were attenuated, indicating a disease driving role of adaptive immunity, specifically CD4+ T cells in MSA pathogenesis [35]. Interestingly, upon further investigation of T cells isolated from Olig001-SYN transduced mice, α-syn overexpression resulted in increased Tbet+ CD4+ T cells population and significant production of the proinflammatory cytokine interferon gamma (IFNγ) [35]. As infiltrating T cells are a significant source of IFNγ, it is not currently known whether IFNγ mediates mechanisms that drive MSA pathogenesis.

IFNγ is an important pro-inflammatory cytokine. In response to proinflammatory stimuli, immune cells such as T cells, myeloid cells, natural killer cells, and B cells can produce IFNγ resulting in MHC mediated antigen presentation [7, 13]. IFNγ released from CD4+ T cells, particularly the Th1 effector subtype, enhances inflammation by binding to its receptor (IFNγR1) and, via the JAK/STAT pathway, activates genes responsible for T cell differentiation, T cell activation, and MHCII antigen presentation. In other demyelinating diseases like multiple sclerosis (MS), IFNγ plays a significant role in disease pathogenesis. In experimental autoimmune encephalomyelitis (EAE), a mouse model for MS, IFNγ neutralizing antibody treatment attenuated neurodegeneration and neuroinflammation [1, 29]. Furthermore, when Tbet (a transcription factor required for CD4+ T cell differentiation into Th1 effector subtypes) was genetically knocked out in mice, it prevented the development of EAE [3] via blocking IFNγ expression. Like MS, IFNγ is significantly increased in the cerebrospinal fluid (CSF) of MSA patients, however, no follow up studies have been conducted to determine if the IFNγ is pathogenic [5, 30]. Despite IFNγ being an important mediator of neuroinflammation in demyelinating disease [2, 29], there are currently no studies investigating a similar role of IFNγ in neuroinflammation, demyelination, and neurodegeneration in MSA.

In this study, we sought to determine the role of IFNγ in α-syn-mediated neuroinflammation, demyelination, and neurodegeneration in the Olig001-SYN mouse model of MSA-P. Utilizing genetic and pharmacological approaches, we found that blocking Tbet expression or neutralizing IFNγ attenuated α-syn mediated neuroinflammation, demyelination, and neurodegeneration. Furthermore, using a novel Thy1.1/IFNγ reporter mouse, we determined that IFNγ was expressed primarily by CD4+ T cells and minimally in other immune cells in response to α-syn overexpression, suggesting that IFNγ producing CD4+ T cells mediate disease pathogenesis in mice. These findings indicate that IFNγ, primarily produced by Th1 T cells, drives neuroinflammation, demyelination, and neurodegeneration in a rodent model of MSA, highlighting IFNγ as a potential therapeutic target for future investigation in MSA.

## Materials and methods

### Mice

Male and female C57BL/6 (#000664 Jackson Laboratories) were used for these studies and maintained on a congenic background. IFNγ/Thy1.1 reporter mice with a C57BL/6 background (generously donated by Dr. Casey Weaver) were also used and have been previously described and characterized [11]. Additionally, male and female Tbet -/- mice (#004648 Jackson Laboratories) were used. Under a C57BL/6 background, these mice have exon 1 of the T-box 21 (*Tbx21*) deleted. All research conducted on animals were approved by the Institutional Animal Care and Use Committee at the University of Alabama at Birmingham (UAB).

### Olig001 vector

The Olig001 vector is a modified AAV capsid generated via directed evolution that has been characterized previously [21, 22, 26]. Briefly, the Olig001 capsid has a > 95% tropism for oligodendrocytes and the vectors utilized contained the CBh promoter and bovine growth hormone polyA, controlling the expression of either transgene (human α-syn or GFP as control).

### Stereotaxic surgery

Male and female mice aged to 8–12 weeks, were anesthetized with isoflurane applied by an isoflurane vaporizing instrument provided by the Animal Resource Program at UAB. Using a Hamilton syringe and an automatic injecting system, mice were unilaterally (luxol fast blue, DAB and immunofluorescence staining; n = 5) or bilaterally (flow cytometry; n = 3–5 and stereology; n = 7–10) injected with 2 μl of Olig001-GFP (1 × 10^13^ vector genomes (vg)/ml) or Olig001-SYN (1 × 10^13^ vg/ml) into the dorsolateral striatum at a rate of 0.5 μl/min to mimic MSA-P. The needle was left in the injection site for an additional 2 min and then slowly retracted over the course of 2 min. The stereotaxic coordinates used from bregma were AP+ 0.7 mm, ML+ / − 2.0 mm, and DV − 2.9 mm from dura. All surgical protocols and aftercare were followed and approved by the Institutional Animal Care and Use Committee at the University of Alabama at Birmingham.

### Immunohistochemistry tissue preparation

Four weeks post transduction of the Olig001 virus, mice were anesthetized and transcardially perfused with 0.01M Phosphate-buffered saline (PBS) pH 7.4, followed by a fixation with 4% paraformaldehyde (in PBS, pH 7.4; PFA). Brains were dissected and incubated in 4% PFA solution for 4 h at 4 °C. After PFA fixation, the brains were cryoprotected in a 30% sucrose (in PBS) solution for 3 days until brains were fully saturated. Brains were frozen and cryosectioned coronally at 40 μm on a sliding microtome. Tissue was stored in a 50% glycerol/PBS solution at − 20 °C.

### Immunofluorescence

Forty-μm thick free-floating sections were washed in 0.01 M tris-buffered solution (TBS; pH 7.4) 3 X for 5 min. The tissue then underwent a sodium citrate antigen retrieval (0.1M; pH 7.3) process for 30 min at 37 °C. After antigen retrieval the free-floating sections were washed and blocked in 5% normal serum for 1–2 h. The sections were thereafter incubated in 1% serum in TBS-Triton (TBST) primary antibody solution consisting of one of the following antibodies: anti-Iba1 (1:500, WACO), anti-Olig2 (1:250, clone SP07-02; R&D), anti-GFAP (1:500, clone DIF48; Abcam), anti-CD3 (1:500, clone 17A2; Thermo Fisher), anti-CD8 (1:500, clone 4SM15; eBioscience), anti-Thy1.1 (1:500, OX-7; Invitrogen) anti-CD4 (1:500, clone RM4-5; Thermo Fisher), anti-NK1.1 (1:250, clone EPR22990-12; Abcam). After an overnight incubation, the free-floating sections were washed and put into a 1% serum TBST secondary solution for 2 h. Sections were mounted onto coated glass slides, and cover slipped using hard set mounting medium (Vector Laboratories). Fluorescence images were collected on a Ti2 Nikon microscope using a Ci2 confocal system.

### DAB labeling and quantification

**Fig. 1:**
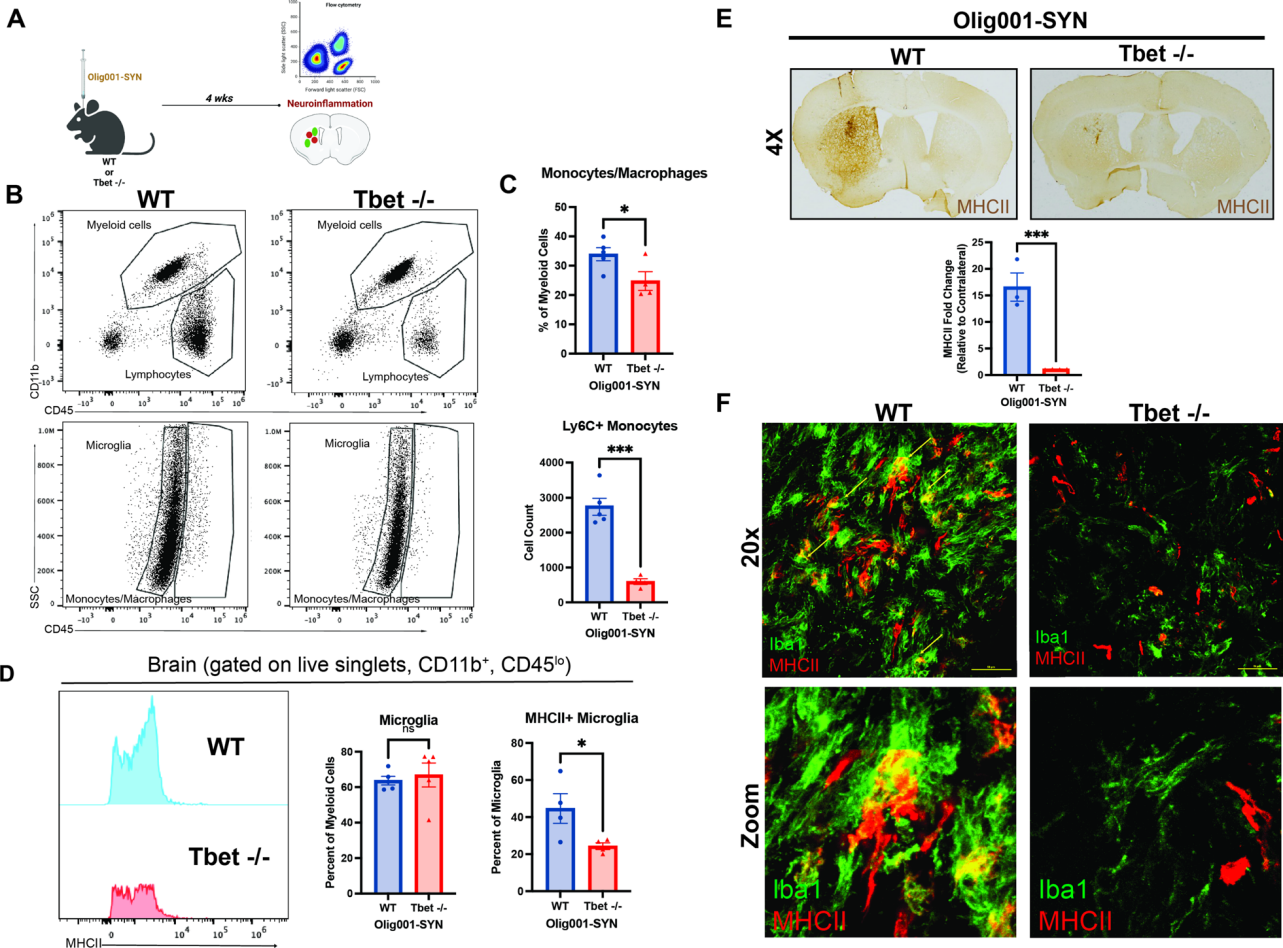
Tbet -/- attenuates myeloid responses in the Olig001-SYN mouse model of MSA. **A** Tbet -/- mice or WT mice 8–12 weeks of age received bilateral (flow cytometry) or unilateral (immunohistochemistry) stereotaxic injections of Olig001-SYN into the dorsal lateral striatum. 4 weeks post-injection, tissue was collected to assess for neuroinflammation. **B** Flow cytometry on isolated striatal tissues, displaying both myeloid (CD45+ , CD11b) and lymphocyte populations (CD45+ , CD11b−) (top); the resident microglia (CD45lo, CD11b +) and monocytes/macrophages (CD45hi, CD11b +) (bottom). **C** The quantification of monocytes/macrophages (CD45hi, CD11b +) and infiltrating monocytes (CD45hi, CD11b+ , Ly6C +) isolated from the striatum with flow cytometry. Mean values ± SEM are plotted, non-parametric Wilcoxon test, *p < 0.05, ***p < 0.001. **D** Quantification of flow cytometry showing the percent of microglia within the striatum and their MHCII expression. Mean values ± SEM are plotted, non-parametric Wilcoxon test, ns = no significance, *p < 0.05. **E** Representative images of 3,3’Diaminobenzidine (DAB) staining and quantification of mean gray value of the MHCII expression in the dorsal lateral striatum. Mean values ± SEM are plotted, non-parametric Wilcoxn test, ***p < 0001. **F** Representative images depicting MHCII expression (red) on activated microglia (Iba1, green) in the dorsal lateral striatum. Scale bars are at 50uM. For immunohistochemistry experiments, n = 3 per group. For flow cytometry experiments to n = 5 (two mouse striatum tissues pooled per n) per group

Free floating striatal sections were quenched in a 3% hydrogen peroxide/50% methanol in 0.01M TBS (pH 7.4) solution at room temp for 5 min. After three TBS washes, tissue was incubated in antigen retrieval sodium citrate solution for 30 min at 37 °C. Background staining was blocked in 5% serum and incubated with either an anti-MHCII (1:500, M5/114.15.2; Thermo Fisher) or a pSer129 (1:5000, clone EPI53644; Abcam) antibody 1% serum overnight at 4 °C. The following day, sections were incubated with a biotinylated goat anti-rat IgG secondary antibody (1:1000, Vector Labs) in a 1% serum TBST solution. The R.T.U Vectastain ABC Reagent kit and DAB kits (Vector Labs) were used to develop the stain according to manufacturer’s protocol. Striatal sections were mounted onto plus coated slides and dehydrated with a gradient of ethanol solutions. Lastly slides were cover slipped with Permount (Electron Microscopy Sciences). Slides were imaged at 10X on a Zeiss imager M2 brightfield microscope (MBF Biosciences). Data was analyzed in ImageJ and the fold change of mean grey value between the ipsi- and contra-lateral side were calculated. This method of analysis was applied to Figs. 1E and Additional file 1: Fig. S1B, S5B.

To quantify pSer129+ cells, sections were immunostained and dehydrated using the DAB protocol described above. After slides were cover slipped, images were taken at 20× on a Zeiss imager M2 brightfield microscope (MBF Biosciences), and pSer129+ cells were counted as previously published [35]. Briefly, images were imported into ImageJ and a grid was overlayed with a 35,000 per point grid. Five grids within the dorsolateral striatum section were chosen at random to count, and the average was calculated. Three sections were counted per slide, and the average GCI count of the three sections was calculated as the total of pSer129+ cells within the dorsolateral striatum of one mouse.

### Interferon gamma neutralizing antibody treatment

C57BL/6 mice were treated three days before Olig001-SYN transduction with i.p. injection of either a neutralizing IFNγ antibody (clone XMG1.2; 200 ng; n = 5) or isotype control (IgG1; 200 ng; n = 5). Three days following the initial i.p. injection, mice received an injection of either Olig001-GFP or Olig001-SYN in the dorsolateral striatum. Immediately after vector injection, mice were given an i.p. dose of their respective treatment. To continue their treatment, mice were injected i.p. every three days with 200 ng of either neutralizing IFNγ or the isotype control. After 30 days, mice were anesthetized for their respected endpoints.

### Luxol fast blue staining and quantification

Forty-μm thick mounted brain sections were quickly washed with DI water and incubated in a 0.1% luxol fast blue solution at 60 °C for 2 h. Excess dye was removed with running DI water. To differentiate and visualize the myelin from the rest of the tissue, slides were dipped in a 0.05% Lithium Carbonate solution two times for 1 min, followed by three 70% ethanol washes. This differentiation step was repeated until the myelin was stained blue and non-lipid parts of the tissue were clear. Slides were quickly dehydrated and mounted with Permount (Electron Microscopy Sciences). Images were taken at 10X on a Zeiss Axio Imager M2 microscope (MFB Biosciences). Images were quantified with ImageJ. For 4 week timepoints, fold change of contra- to ispi-lateral were calculated based on mean grey value of the luxol stain in the striatum and corpus callosum. Due to the bilateral injections of the 6-month timepoint, the mean grey value of Olig001-SYN and Olig001-GFP were calculated. and the fold change was calculated by comparing the dorsal striatum and corpus callosum of Olig001-SYN to Olig001-GFP.

### Mononuclear cell sorting and flow cytometry

Four weeks post Olig001 delivery, mice were anesthetized and transcardially perfused with 0.01M PBS pH 7.4. Brain tissue was removed and the striata were dissected. Striatal tissues were triturated and digested with 1 mg/mL Collagenase IV (Sigma) and 20 μg/mL DNAse I (Sigma) diluted in RPMI 1640 with 10% heat inactivated fetal bovine serum, 1% glutamine (Sigma), and 1% Penicillin–Streptomycin (Sigma). After enzyme digestion, samples were filtered through a 70 uM filter and mononuclear cells were separated out using a 30/70% percoll gradient (GE).

For all cell labeling, isolated cells were blocked with anti-Fcy receptor (1:100; BD Biosciences). Cell surfaces were labeled with the following fluorescent-conjugated antibodies against CD45 (clone 30-F11; eBioscience), CD11b (clone M1/70; BioLegend), MHCII (clone M5/114.15.2; BioLegend), Ly6C (clone HK1.4; BioLegend), CD4 (clone GK1.5; BioLegend), CD8a (clone 53.6.7; BioLegend), Thy1.1 (clone HIS51; BD Biosciences), anti-PDGFRa (clone APA5; BioLegend), anti-O4 (R&D Systems), or hCD2 (clone RPA2.10; eBioscience). A fixable viability dye was used to distinguish live cells per manufacturer’s instructions (Fixable Near-IR LIVE/DEAD Stain Kit, Invitrogen).

For intracellular transcription factor labeling, the Foxp3/Transcription Factor Staining Kit (eBioscience) was used accordingly with fluorescent-conjugated antibodies against FOXP3 (clone FJK-16S; eBioscience), T-bet (clone 4B10; BioLegend), GATA2 (clone 16E10A23; BioLegend), RORγt (clone Q31-378; BD Biosciences), or Olig2 (clone 211F1.1; Sigma Millipore). An Attune Nxt (Thermo Fisher Scientific) or a BD Symphony flow cytometer (BD Sciences) were used to analyze samples and FlowJo (Tree Star) software were used for analysis. Mean cell count numbers, percentages, and mean fluorescent intensity (MFI) were measured with FlowJo software to assess for neuroinflammation.

### Stereology analysis for striatum

To quantify NeuN+ neurons within the dorsolateral striatum, unbiased stereology was used. Mice used for stereological assessment were bilaterally injected for behavioral time points. Six months post transduction, tissues were harvested and sectioned as described in the immunohistochemistry tissue preparation section above. Free floating sections were stained with NeuN (clone EPR12763; Abcam) and developed with the DAB protocol described above. Once slides were stained and cover slipped, Zeiss Axio Imager M2 microscope and StereoInvestigator Software (version 2021.1.3) (MFB Biosciences) was used to count NeuN+ cells. Due to the localization of α-syn, the contours used for counting were drawn around the dorsolateral striatum (Fig. 4A). To encompass areas of pathology, six serial sections were counted per mouse. Grid size was 300 μm × 300 μm and the counting frame was set at 40 × 40. After dehydration, the thickness of the sections was determined to be 25 μm. Six sections of the striatum were counted and the average counting frame per section was 23. Estimated populations were generated, and a two-way ANOVA and a Tukey post hoc test was used to determine the neuronal loss significance.

### Stereology analysis for substantia nigra pars compacta

The TH+ neurons in the substantia nigra pars compacta (SNpc) were quantified using methods that were previously described [10, 36]. Briefly, SNpc tissue sections were stained with anti-tyrosine hydroxylase (TH) (EP1536Y, Abcam) using the DAB labeling and quantification protocol as described. Once tissue was stained and mounted, the same scope and software used in the stereology for NeuN+ counting was used to analyze TH+ neurons. Five sections were counted per mouse. Grid size of 100 μm × 100 μm with a counting frame of 50 μm × 50 μm was used to count TH+ neurons. The thickness of the sections was 25 μm. Estimated populations were generated and compared to a naïve control. Data was analyzed with a one-way ANOVA with Tukey post test for significance (with 95% confidence and *p* < 0.05).

### Open Field

At 6 months post Olig001-GFP or Olig001-SYN induction, mice were assessed for motor deficits with open field. Before testing, mice were habituated to testing room for 1 h at the start of the day. Mice were placed in a 44.45 cm × 44.45 cm plastic box and the Noldus Ethovision XT 16 software recorded the activity of the mice for 15 min. After the 15 min, mice were returned to their home cage. The total distance and velocity were measured and recorded.

### Statistical analysis

All graphs and corresponding statistical tests were generated or performed using Prism software (GraphPad). For flow cytometry, data points were compared across time points/antibody treatment using a ranked-summed, non-parametric Wilcoxon test (with 95% confidence and *p* < 0.05). Stereology analysis in Fig. 4 was analyzed with a two-way ANOVA was used and Tukey’s Honestly-Significant-Difference (with 95% confidence and *p* < 0.05) was used to determine significance. Stereology analyzed in Additional file 1: Fig. S3 with a one-way ANOVA with Tukey posthoc test for significance (with 95% confidence and *p* < 0.05).

### Data availability

The authors affirm that the findings of this manuscript are supported by the data therein. Additional information can be requested from the corresponding author.

## Results

### Genetically deleting Tbet attenuates neuroinflammation in the Olig001-SYN mouse model of MSA

IFNγ signaling induces MHCII expression on the cell surface of APCs [13]. To determine if α-syn overexpression in oligodendrocytes induced neuroinflammation as a result of IFNγ expression, we utilized mice in which the required transcription factor for IFNγ production in Th1 cells, Tbet, was deleted (Tbet -/-). Without Tbet, a Th1 cell cannot carry out its effector functions, one of those being producing IFNγ. Tbet -/- and WT mice, aged 8–12 weeks, received an intracranial injection of 2uL of Olig001-SYN (or Olig001-GFP as control) in the dorsolateral striatum (Fig. 1A). 4 weeks post transduction, using immunohistochemistry (IHC) and mononuclear cell isolation and flow cytometry, we found that Tbet -/- mice showed a reduction in infiltrating Ly6C+ monocytes (CD11b+ , CD45hi, Ly6C+) (Fig. 1B and C). Within the monocyte population there was no difference in MHCII expression (Fig. 1C). In response to α-syn expression, Tbet -/- mice displayed no change in the number of microglia within the striatum (Fig. 1C), however, in the absence of Tbet, a significant reduction in MHCII expression was observed on the cell surface of microglia (CD11b+ CD45lo) via flow cytometry (Fig. 1D). Consistent with our flow cytometry results, utilizing immunohistochemistry, we observed a decrease in MHCII expression in the dorsolateral striatum (Fig. 1E and F) indicating Tbet expression is required for MHCII expression and peripheral monocyte entry.

**Fig. 2:**
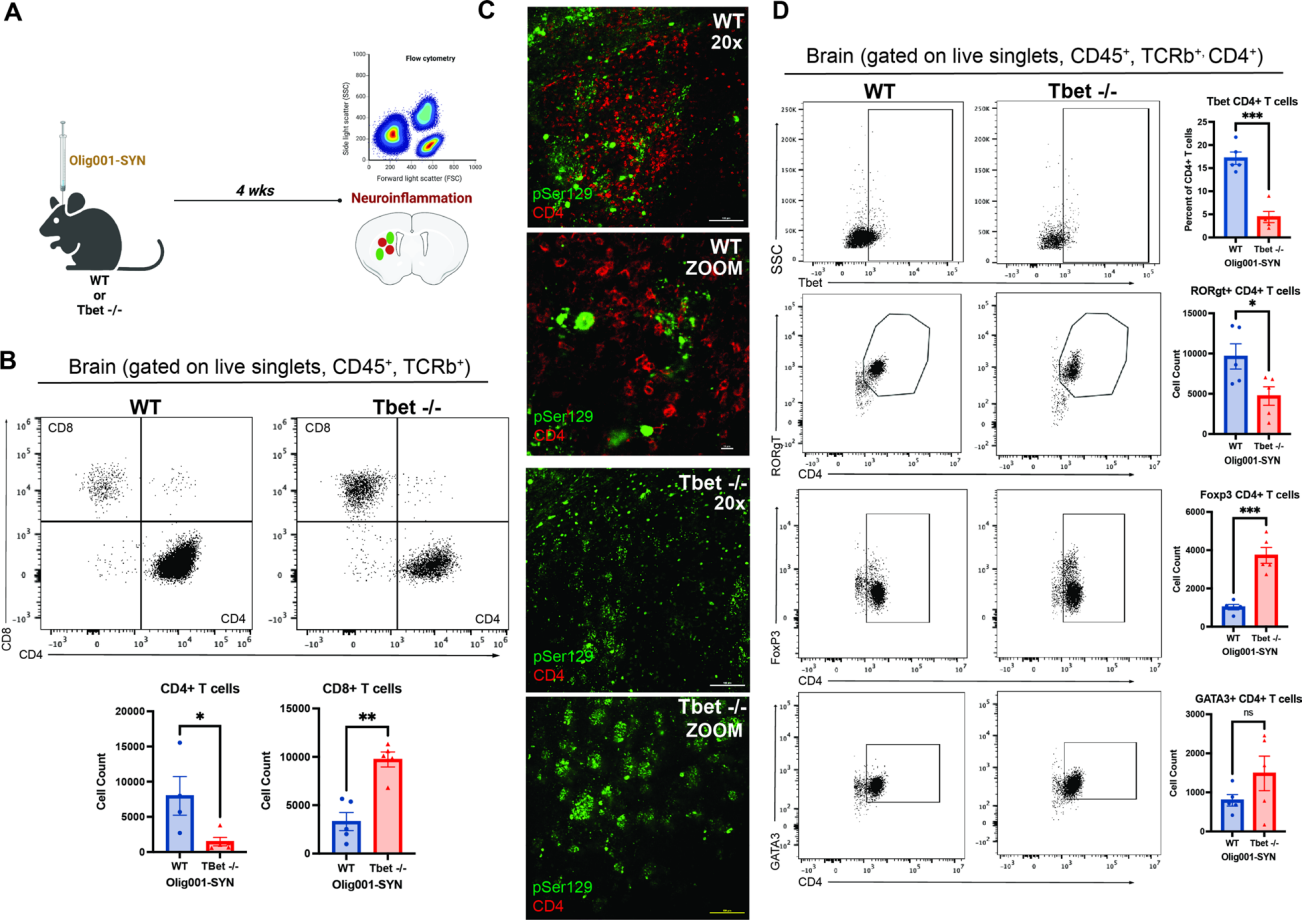
Tbet -/- attenuates CD4+ T cell infiltration and alters T cell effector subsets. **A** At 8–12 weeks, Tbet -/- or WT controls were stereotactically injected with Olig001-SYN into the dorsal lateral striatum. After 4 weeks, tissue was harvested to assess for neuroinflammation. **B** Flow cytometry dot plots of CD8+ (CD45+ , CD11b−, TCRb+ , CD8 +) and CD4+ T cells (CD45+ , CD11b−, TCRb+ , CD4 +). Below the dot plots are the cell counts of CD4+ and CD8+ T cells in the striatum in the presence of α-syn pathology within oligodendrocytes. Mean values ± SEM are plotted ± , non-parametric Wilcoxon, *p < 0.05, **p < 0.01. **C** Representative immunohistochemistry images of pSer129 (green) and CD4+ (red) in the dorsolateral striatum of Tbet -/- mice and WT controls. Scale bars are at 50uM. **D** Flow cytometry graphs and their corresponding quantification for the following CD4+ T cell subsets: Th1, Th17, T_reg_, Th2. Mean values ± SEM are plotted, non-parametric Wilcoxon test, ns = no significance, *p < 0.05, ***p < 0.001. For immunohistochemistry experiments, n = 3 per group. For flow cytometry experiments n = 5 (two mouse striatum tissues pooled per n) per group

To determine whether Tbet-mediated IFNγ is responsible for T cell infiltration in response to α-syn overexpression, we performed mononuclear cell isolation and flow cytometry 4 weeks post vector delivery in WT and Tbet -/- mice (Fig. 2A). Tbet -/- mice displayed a significant reduction in infiltration of CD4+ T cells (Fig. 2B) and an increase of CD8+ T cells (Fig. 2B). Given our previous observations showing that CD4+ T cells are required for MSA pathology, we further investigated the CD4+ T cell response. In the dorsolateral striatum, compared to WT mice, Tbet -/- mice displayed a reduction of CD4+ T cells in close proximity to α-syn (pSer129+) GCI pathology (Fig. 2C). Within the CD4+ T cell population, there were significant changes among the T cell effector subsets Th1, Th17, and T_reg_ including a reduction in the number of Th1 and Th17 cells (Fig. 2D). Conversely, there was a significant increase in the number of T_reg_ cells within the CD4+ T cell population, suggesting that loss of Tbet shifts the T cell repertoire from a pro-inflammatory (Th1, Th17) to a restorative (T_reg_) state. As IL-17 produced by T cells can also contribute to proinflammatory or autoimmune responses [15, 16, 20], we sought to determine if IL-17 producing T cells, Th17, were contributing to disease pathogenesis. To further understand Th17 cells, we utilized mice deficient in the transcription factor required for Th17 development, RORγT -/-. We performed IHC and flow cytometry on WT and RORγT -/- treated with Olig001-SYN. Four weeks post-transduction, RORγT -/- mice overexpressing α-syn in oligodendrocytes showed no attenuation of MHCII expression, CD4+ and CD8+ T cell entry, and enhanced demyelination compared to WT mice (Additional file 1: Fig. S1) indicating that IL-17 producing Th17 cells do not drive Olig001-SYN mediated neuroinflammation and demyelination.

### Tbet expression mediates demyelination in the Olig001-SYN mouse model of MSA

**Fig. 3:**
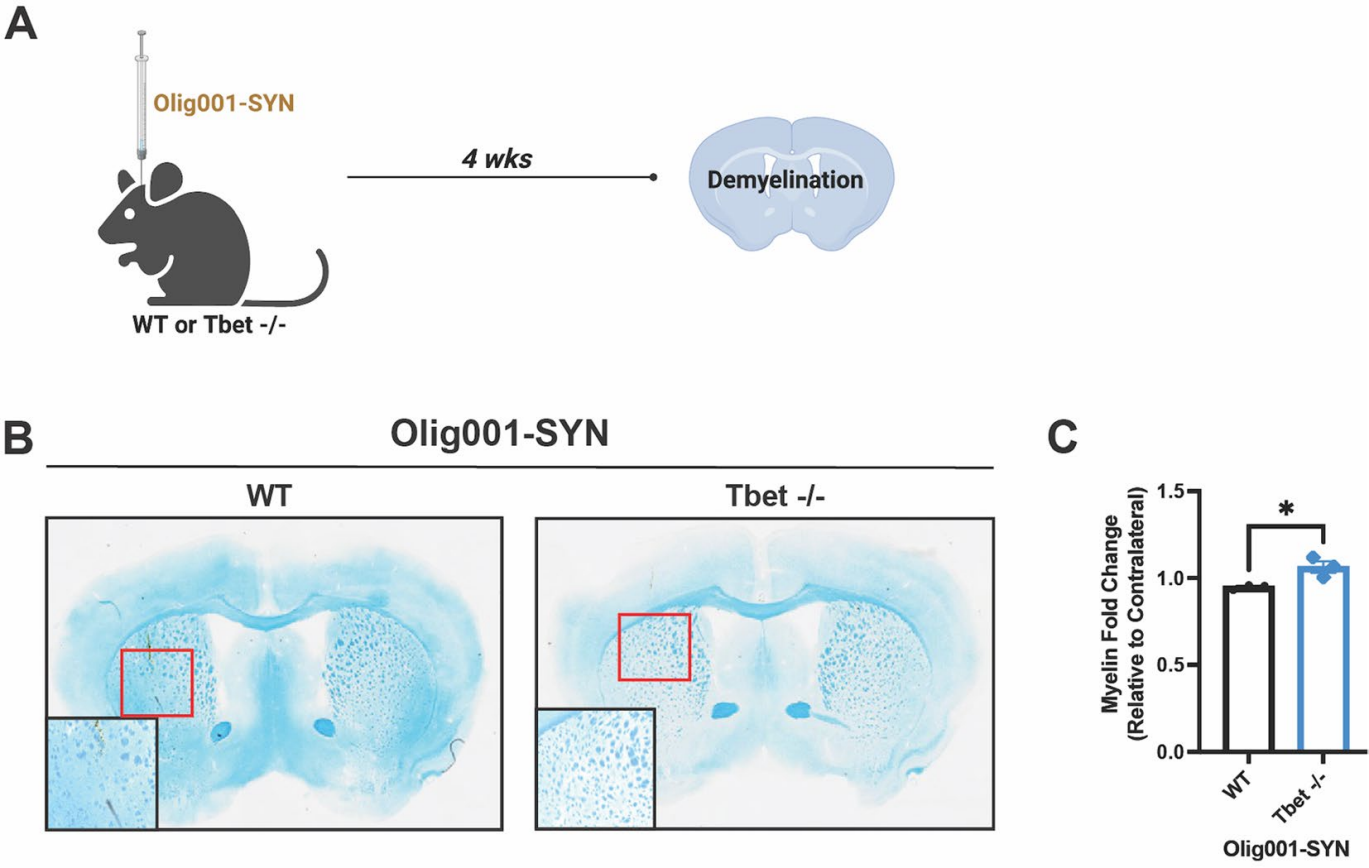
Tbet -/- attenuates Olig001-SYN mediated demyelination. **A** Both male and female Tbet -/- mice and WT controls were transduced with Olig001-SYN at 8–12 weeks old. 4 weeks post transduction, tissue was collected and stained with luxol fast blue to determine demyelination in the striatum and corpus collosum. **B** Representative luxol fast blue images of WT and Tbet -/- where myelinated areas are stained in blue. Bottom left boxes are zoomed areas of demyelination that is highlighted in the red boxes. (**C**) Quantification of the myelin fold change between the ispi- and contralateral sides of the striatum and corpus collosum in WT and Tbet -/- mice. Mean values ± SEM are plotted, non-parametric Wilcoxon test, *p < 0.05. For immunohistochemistry experiments, n = 3 per group; each data point represents a mouse

Demyelination is a key feature of MSA pathology [8] and is observed 4 weeks post transduction in the Olig001-SYN mouse model of MSA. To assess how Tbet contributes to this pathology, Tbet -/- and WT controls, 8–12 weeks of age, were injected with Olig001-SYN in the dorsolateral striatum (Fig. 3A). 4 weeks post-transduction demyelination was assessed via luxol fast blue staining. As a control, naïve, WT and Tbet -/- mice were assessed for luxol fast blue staining and flow cytometry to determine whether Tbet expression affects myelin integrity and oligodendrocyte cell numbers in the striatum. No significant differences were observed between WT and Tbet -/- mice prior to Olig001 induction (Additional file 1: Fig. S2E–G). Following Olig001-SYN transduction, when compared to WT mice, Tbet -/- mice displayed a fourfold increase in the degree of myelination in the dorsolateral striatum and corpus callosum (Fig. 3B and C). The preservation of myelin seen in Tbet -/- is comparable to healthy, non-inflamed mouse striatum (Fig. 3B) indicating that Tbet expression is required for demyelination in the Olig001-SYN mouse model of MSA.

### Tbet expression is required for Olig001-SYN mediated demyelination and neurodegeneration

**Fig. 4:**
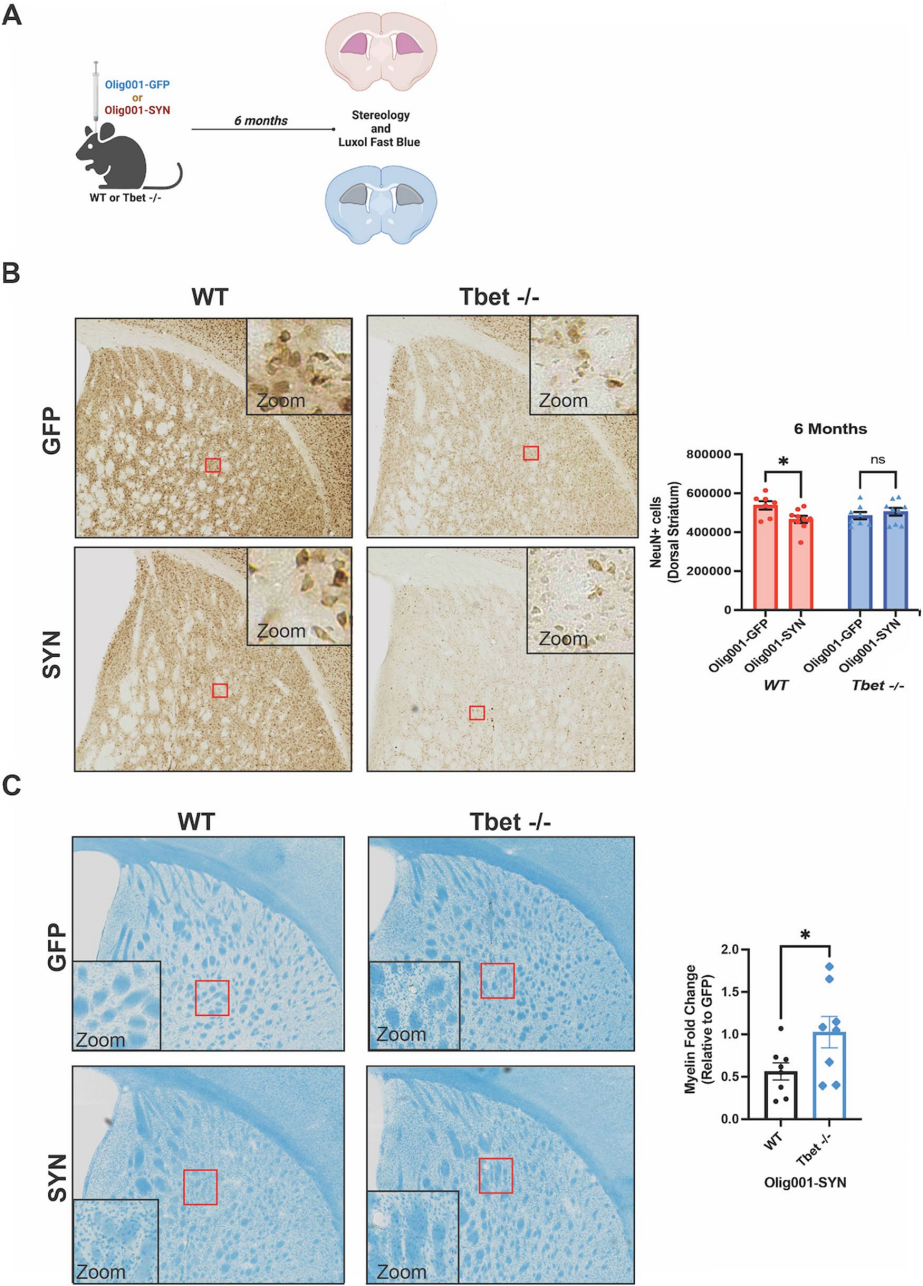
Tbet -/- mice are protected against neuronal loss and demyelination in the presence of α-syn GCI pathology. **A** WT and Tbet -/- were injected bilaterally with either Olig001-GFP or Olig001-SYN into the dorsal striatum. Pathology developed over 6 months, and then tissue was collected for unbiased stereology and luxol fast blue staining. The contours on the tissue cross section define the area of striatum that was quantified for both stereology and luxol fast blue. **B** Representative images of NeuN+ stains used in unbiased stereology analysis. The red boxes highlight the area where the zoom insert (top right) was taken from. Quantification of the unbiased stereology is to the right of the representative images. Mean values ± SEM are plotted, two-way ANOVA with Tukey post hoc for significance, *p < 0.05. **C** Representative images of the luxol fast blue stain used to assess for demyelination. The red boxes highlight the area where the zoom insert (bottom left) was taken from. Quantification is to the right of images. Mean values ± SEM are plotted, student *t*-test, *p < 0.05. For unbiased stereology and luxol fast blue, n = 7–9 per group. Each data point represents one mouse

Previous studies have indicated α-syn overexpression in oligodendroglia results in neurodegeneration in the dorsolateral striatum of rats [22]. Additionally, there is NeuN+ cell loss within the dorsolateral striatum of mice when α-syn is overexpressed in oligodendroglia (Additional file 1: Fig. S3). To determine whether IFNγ drives α-syn -induced neurodegeneration, mice were bilaterally injected with either Olig001-GFP or Olig001-SYN. 6 months post transduction, tissue was collected and unbiased stereology was conducted in the dorsolateral striatum (Fig. 4A). Preliminary analysis in 3 month old naïve Tbet -/- mice indicated a significant reduction in the number of NeuN+ cells in the dorsolateral stratum compared to WT mice (Additional file 1: Fig. S2B). 6 months post transduction, WT mice transduced with Olig001-SYN displayed a significant loss of NeuN+ neurons in the dorsolateral striatum (Fig. 4B). However, when compared to the Olig001-GFP control, Tbet -/- mice displayed no significant NeuN+ cell loss between the Olig001-GFP and Olig001-SYN groups (Fig. 4B) indicating Tbet mediates Olig001-SYN induced neurodegeneration. To control for Olig001 vector toxicity, an additional 6-month study was conducted in WT mice transduced with either Olig001-GFP or Olig001-SYN (Additional file 1: Fig. S3). we observed no significant cell loss in the dorsal-lateral striatum between WT naïve and WT Olig001-GFP mice, 6 months post transduction (Additional file 1: Fig. S3B and C).

To determine if α-syn overexpression in oligodendroglia results in TH+ neuron loss in the SNpc, serial sections from WT Olig001-GFP and Olig001-SYN mice were analyzed via unbiased stereology at 6 months post transduction (Additional file 1: Fig. S3D). No significant TH+ cell loss was observed in WT mice transduced with Olig001-GFP control or Olig001-SYN (Additional file 1: Fig. S3D). At 6 months post-transduction, significant neuroinflammation was observed in WT Olig001-SYN injected mice including persistent myeloid activation and infiltrating T cells (Additional file 1: Fig. S4).

To understand if IFNγ drives long term demyelination similar to what is seen in post-mortem MSA tissue, demyelination was assessed at 6 months with a luxol fast blue stain. We observed no change in myelin within the Tbet -/- Olig001-SYN group when compared to, Olig001-GFP control, (Fig. 4C), whereas the WT Olig001-SYN group showed significant demyelination when compared to WT Olig001-GFP (Fig. 4C). Together, these results (Fig. 4B and C) support the hypothesis that IFNγ is crucial neuroinflammation, demyelination, and neurodegeneration in the Olig001-SYN mouse model of MSA.

### Pharmacologically targeting IFNγ attenuates Olig001-SYN mediated neuroinflammation

**Fig. 5:**
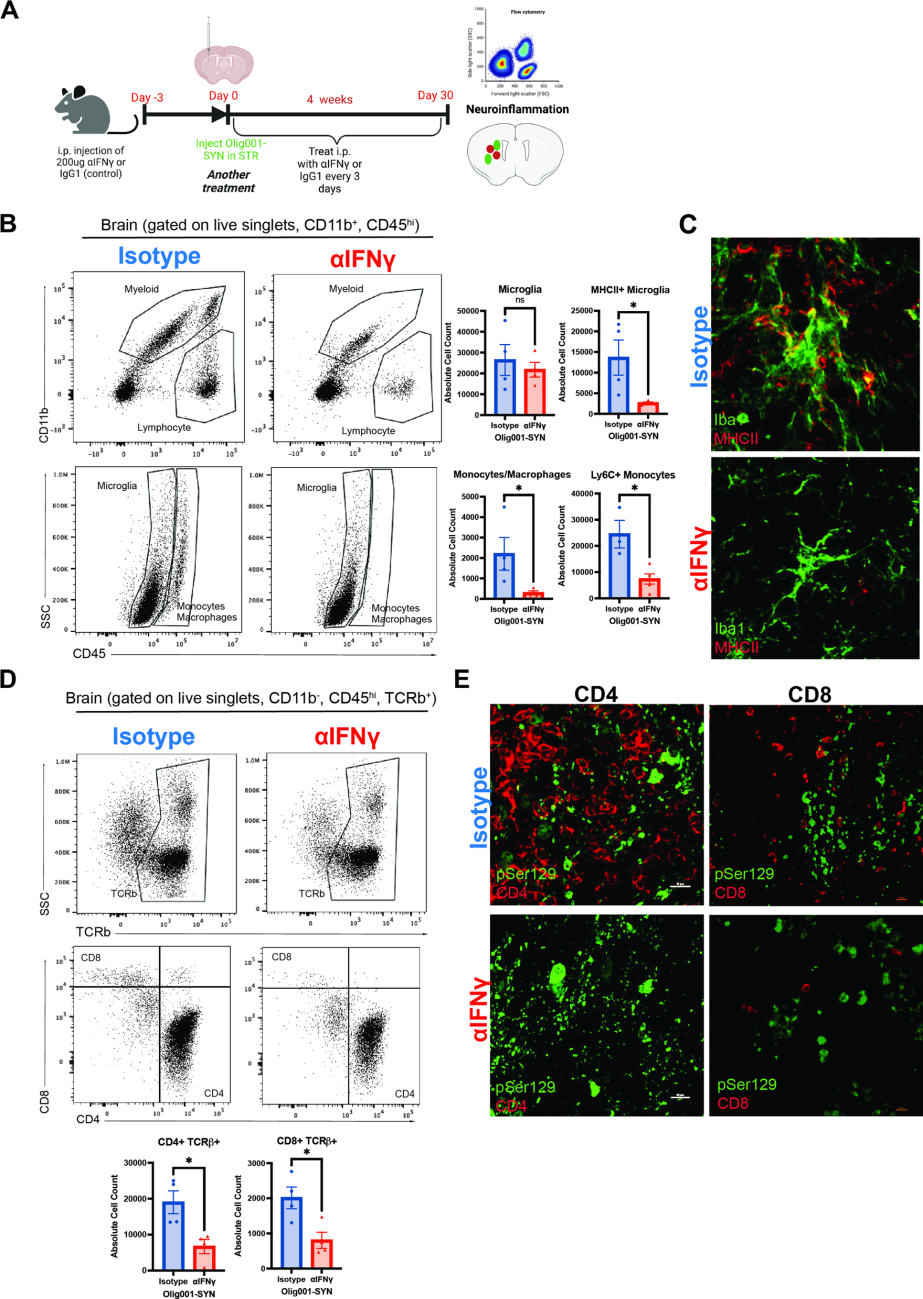
Neutralizing IFNγ activity attenuates neuroinflammation. A Three days prior to Olig001-SYN transduction, both WT female and male 8–12 week old mice received an i.p. injection of 200ng of anti-IFNγ (XMG1.2) or its isotype control (IgG1). Three days later, Olig001-SYN was transduced into the striatum and received another treatment. Afterwards mice received follow up treatments every three days for 30 days. After the 30 days, tissue was collected to assess for neuroinflammation. **B** Dot plots displaying the overall myeloid populations (CD45+ , CD11b +) and lymphocytes (CD45+ , CD11b−). Below are dot plots of microglia (CD45^mid^, CD11b +) and monocytes/macrophages (CD45^hi^, CD11b +). Mean values ± SEM are plotted, non-parametric Wilcoxon test, ns = no significance, *p < 0.05. **C** Representative immunohistochemistry of Iba1 (green) and MHCII (red) positive cells within the striatum and corpus collosum. **D** flow cytometry of mononuclear TCRb+ (CD45+ . CD11b−, TCRb +), CD4+ (CD45+ , CD11b−, TCRb +) and CD8+ T cells (CD45+ , CD11b−, TCRb +). Mean values ± SEM are plotted, non-parametric Wilcoxon *p < 0.05. **E** representative immunohistochemistry images of CD4+ and CD8+ T cells (red) and insoluble α-syn, pSer129 (green) in the striatum. Scale bars are at 10uM. For immunohistochemistry experiments, n = 3 per group. For flow cytometry experiments n = 4 (two mouse striatum tissues pooled per n) per group

Our genetic approach established a role for Tbet-mediated IFNγ in Olig001-SYN-mediated MSA pathology. However, as IFNγ expression is not the only pathway mediated by Tbet, a key question remains: is IFNγ driving pathology in the Olig001-SYN model? To specifically determine the role of IFNγ in Olig001-SYN mediated neuroinflammation and demyelination, an IFNγ neutralizing antibody was used to globally decrease IFNγ. WT mice (8–12 weeks old) were pre-treated with either an IFNγ neutralizing antibody (XMG1.2; 200ng) or an isotype control (IgG1; 200ng) intraperitoneally (i.p.) three days prior to Olig001-SYN transduction, and every three days over the course of 4 weeks (Fig. 5A). Four weeks post-transduction, striatal tissue was harvested to assess for neuroinflammation via immunohistochemistry and flow cytometry. Our results indicate XMG1.2 treatment significantly attenuated pro-inflammatory monocyte infiltration and MHCII expression on microglia (Fig. 5B and C) in the ipsilateral striatum compared to isotype control. Additionally, XMG1.2 treatment significantly decreased the number of CD4+ and CD8+ T cells infiltrating the ipsilateral striatum (Fig. 5D). While α-syn expression in oligodendrocytes was unaffected by XMG1.2 treatment (Fig. 5E, Additional file 1: Fig. S5), peripheral T cell entry was attenuated confirming that IFNγ is mediating α-syn-induced neuroinflammation.

### Pharmacologically targeting IFNγ attenuates Olig001-SYN mediated demyelination

**Fig. 6:**
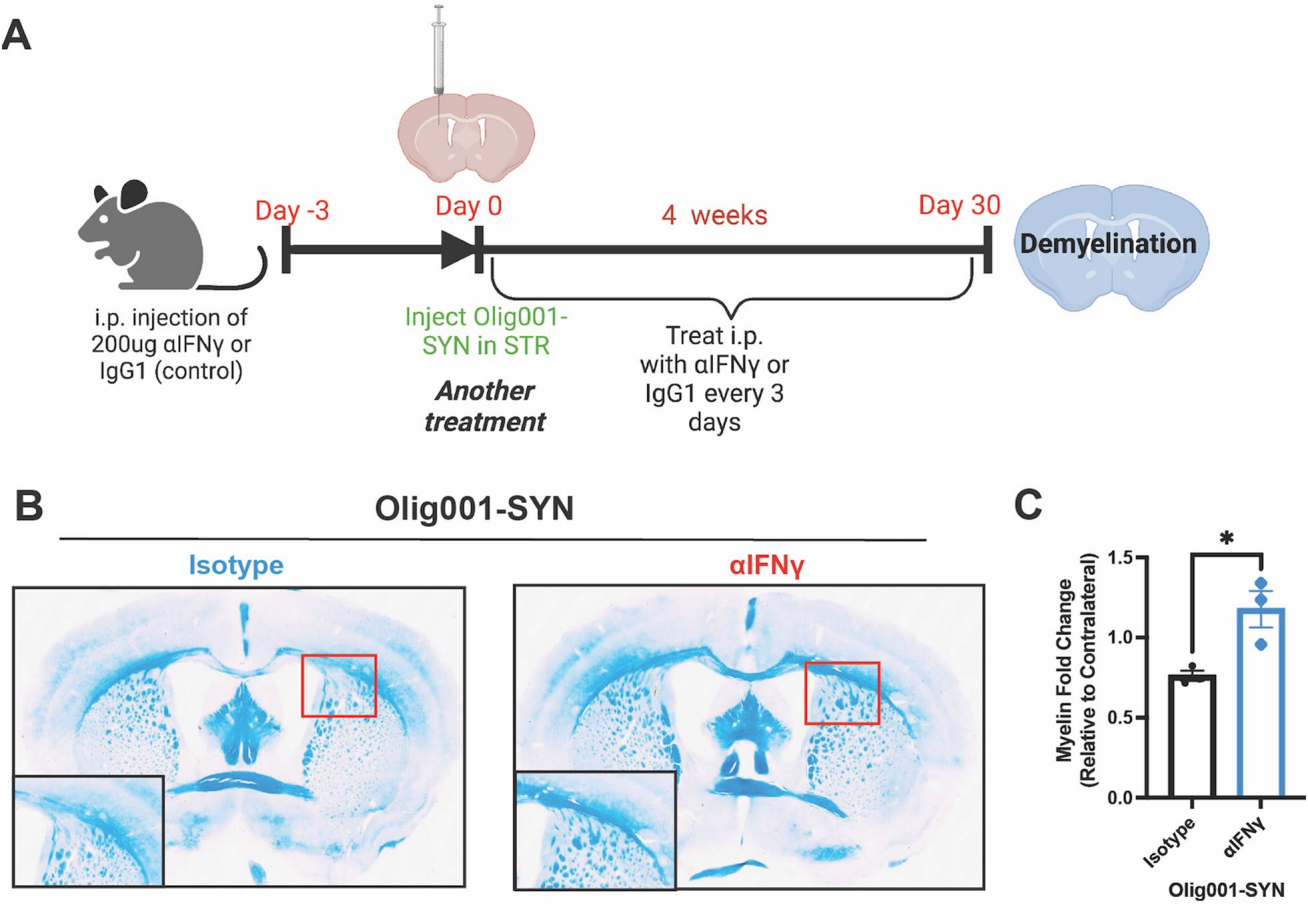
Neutralizing IFNγ attenuates Olig001-SYN mediated demyelination. **A** Same experiment design that was used in assessing neuroinflammation in Fig. 5. **B** Representative images of luxol fast blue staining displaying myelin within the striatum. Boxes in the bottom left are zooms of areas of demyelination highlighted in red. Red arrows point to areas of demyelination. (C) Myelin fold change between ipsi- and contra-lateral sides of the striatum and corpus collosum. Mean values ± SEM are plotted, non-parametric Wilcoxon test, *p < 0.05. For immunohistochemistry experiments, n = 3 per group. Each data point represents one mouse

Lastly, to determine if IFNγ drives demyelination in Olig001-SYN, luxol fast blue staining was performed on striatal sections from Olig001-SYN treated mice (Fig. 6A). XMG1.2 treatment preserved myelin in the dorsolateral striatum, specifically in the corpus callosum, compared to isotype control in Olig001-SYN transduced mice (Fig. 6B and C). These results indicate that neutralizing IFNγ attenuates Olig001-SYN mediated demyelination, again supporting a role of IFNγ expression in Olig001-SYN-mediated demyelination.

### CD4+ T cells produce IFNγ in response to Olig001-SYN mouse model

**Fig. 7:**
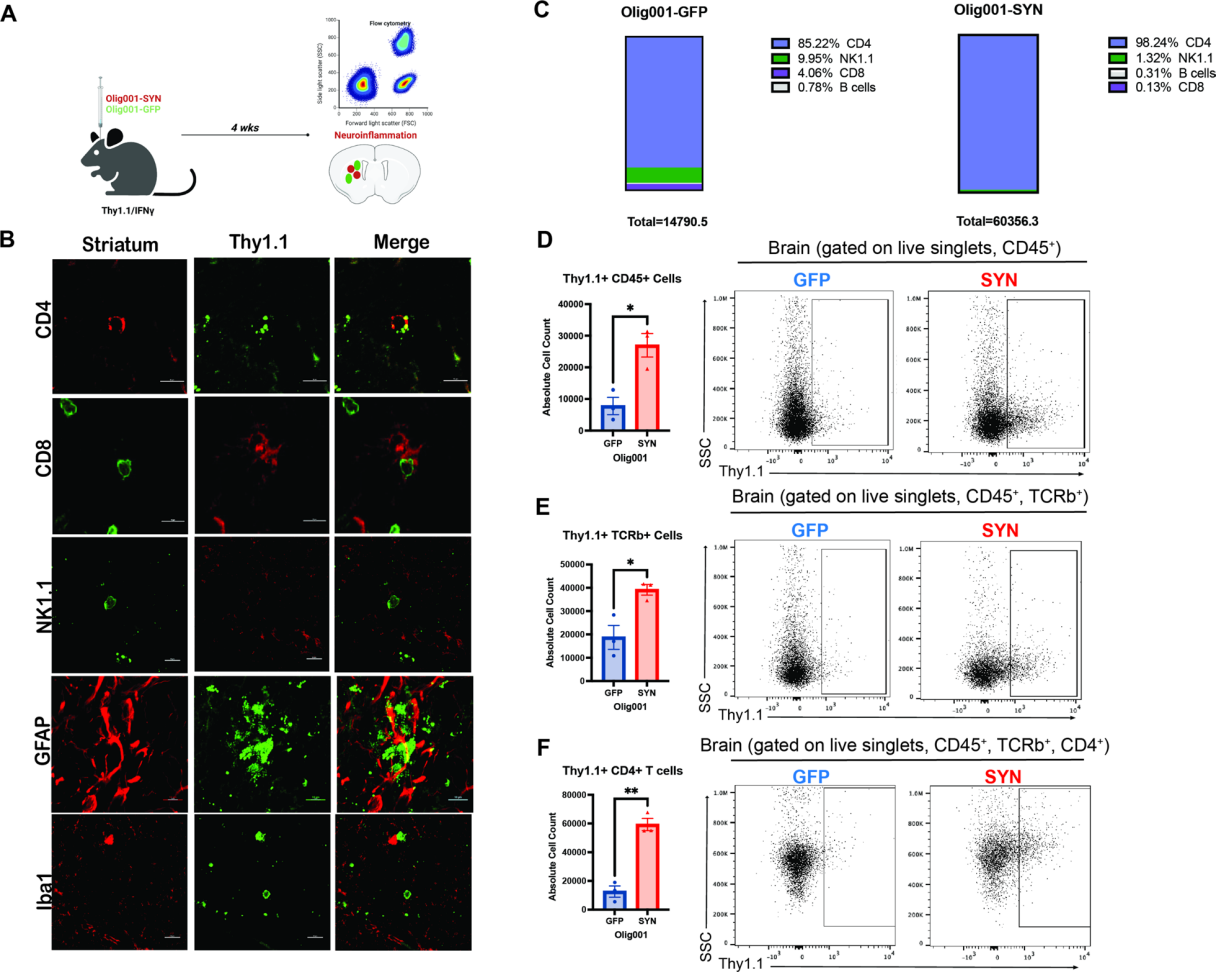
IFNγ is produced primarily by CD4+ T cells. **A** Male and female 8–12-week-old Thy1.1/IFNγ reporter mice were injected with either Olig001-GFP or Olig001-SYN in the dorsal lateral striatum. 4 weeks post-transduction, tissue was harvested for neuroinflammation. **B** A panel of representative immunohistochemistry images of CD4+ T cells (CD4), CD8+ T cells (CD8), NK cells (NK1.1), astrocytes (GFAP), and microglia (Iba1) with the IFNγ reporter Thy1.1. **C** Percentages of CD45+ cells generated from flow cytometry data looking at Thy1.1+ (IFNγ producing) cells. **D** Dot plots between Olig001-GFP and Olig001-SYN injected Thy1.1/IFNγ reporter mice showing Thy1.1+ cells in lymphocytes (CD45+ , CD11b−). Mean values are plotted ± SEM, non-parametric Wilcoxon, *p < 0.05. **E** Flow cytometry dot plots of Thy1.1+ TCRb+ T cells (CD45+ , CD11b−, TCRb+ , Thy1.1 +). Mean values are plotted ± SEM, non-parametric Wilcoxon test, *p < 0.05. **F** Flow cytometry showing Thy1.1+ CD4+ T cells (CD45+ , CD11b−, TCRb+ , CD4+ , Thy1.1 +). Mean values are plotted ± SEM, non-parametric Wilcoxon test, **p < 0.01. For immunohistochemistry experiments, n = 3 per group. For flow cytometry experiments n = 3 (two mouse striatum tissues pooled per n) per group

In post-mortem MSA tissue there is evidence of infiltrating CD4+ and CD8+ T cells [35] in the parenchyma and increased IFNγ in the CSF [5], and previous studies in preclinical models have shown that CD4+ T cells are required for neuroinflammation and demyelination [35]. Similarly, the results presented here, using genetic and pharmacological approaches, show that IFNγ is a key mediator of the neuroinflammation, demyelination, and neurodegeneration observed in the Olig001-SYN model. While our previous studies in the Olig001-SYN model suggest IFNγ is produced by T cells [35], it is unclear if other CNS resident and infiltrating immune cells produce IFNγ in response to α-syn expression in oligodendrocytes. To determine which CNS resident and infiltrating immune cells express IFNγ as a result of α-syn overexpression, we used a Thy1.1/IFNγ reporter mouse where Thy1.1 is expressed from the IFNγ promoter [11]. In this mouse, when IFNγ is expressed, Thy1.1 is expressed on the cell surface. Using this reporter model, we isolated mononuclear cells from the dorsolateral striatum of mice treated with Olig001-SYN or Olig001-GFP control (Fig. 7A). Using immunohistochemistry, immune populations known to produce IFNγ (CD4+ T cells, CD8+ T cells, NK cells, astrocytes, and microglia) were investigated for Thy1.1 expression on the cell surface via IHC and flow cytometry (Fig. 7B). These lymphocytes (CD45+) were analyzed, and our results indicate that CD4+ T cells expressed the overwhelming majority of Thy1.1 on the cell surface in response to α-syn overexpression in oligodendrocytes (Fig. 7C). Upon further investigation, the majority of Thy1.1 expression was found on CD45+ TCRb+ CD4+ T cells (Fig. 7D–F), matching results seen in IHC experiments (Fig. 7B). Given that CD4+ T cells are producing the pathogenic IFNγ (Fig. 7F) when α-syn is expressed in oligodendrocytes, these data suggest that the CD4+ T cell effector subtype, Th1 cells, are facilitating the disease process via production of IFNγ. Together, our results show that other immune cell types like CD8+ T cells, B cells, and NK cells do not significantly express IFNγ following α-syn overexpression in oligodendrocytes, but CD4+ T cells drive MSA pathology via IFNγ expression.

## Discussion

In this study we show that IFNγ, primarily produced by CD4+ T cells, drives neuroinflammation and demyelination in the Olig001-SYN mouse model of MSA. Using a genetic approach in which Tbet, the primary transcription factor necessary for IFNγ expression, was globally knocked out, neuroinflammation and demyelination were attenuated at four weeks post vector delivery. At 6 months post Olig001-SYN induction, neuronal loss was attenuated in Tbet -/- mice indicating Tbet-mediated IFNγ expression is also required for Olig001-SYN-mediated neurodegeneration. To understand if IFNγ was the primary cytokine driving disease pathology, IFNγ was targeted via IFNγ neutralizing antibody (XMG 1.2). When IFNγ was neutralized, there was a significant decrease in activated CNS myeloid populations, MHCII expression, and infiltrating CD4+ and CD8+ T cells. Lastly, using a novel IFNγ reporter mouse, we found that the Olig001-SYN-mediated increase in IFNγ expression originates from CD4+ T cells, suggesting that Th1, not Th17 T cells, are key in facilitating neuroinflammation, demyelination, and neurodegeneration. Overall, our results indicate that IFNγ mediates α-syn-mediated neuroinflammation, demyelination, and neurodegeneration in a mouse model of MSA, and thus targeting IFNγ-producing CD4+ T cells is likely to be disease modifying.

In this study, Tbet deficiency resulted in significant attenuation of CD4+ T cell infiltration, CNS myeloid activation, and infiltrating monocytes, indicating that genetically blocking IFNγ expression attenuates Olig001-SYN mediated neuroinflammation (Figs. 1, 2, 3 and 4). While genetic deletion of Tbet attenuated CD4+ T cell infiltration and shifted the T cell repertoire in favor of T_reg_ cells, interestingly, there was a significant increase in CD8+ T cells. Although unexpected, this observation highlights either a compensatory effect of Tbet deficiency or a possible role of CD8+ T_regs_ in repressing an inflammatory response. Growing evidence in human and mouse MS studies highlight the existence of CD8+ T_regs_, and their role in suppressing proinflammatory cells like Th1 and Th17 [15, 16, 18]. In human post-mortem MSA tissue, our previous study showed a significant presence of CD8+ T cells [35]. However, studies in blood have shown a decrease in CD8+ T cells and a significant shift in the CD4/CD8 ratio favoring CD4+ T cells [4]. While our previous studies have shown CD4+ T cells are important in disease progression, there is little evidence in the literature investigating the role of CD8+ T cells in MSA. Further studies are needed to understand the role of CD8+ T cells in MSA and whether they mediate neuroinflammatory and neurodegenerative responses via their cytotoxic effector functions.

Although there has been extensive research into other demyelinating disease like MS, the mechanism of how demyelination occurs is still unclear [28]. Demyelination and neurodegeneration are pathologies that are evident in MSA post-mortem tissue and studies have shown demyelination occurs before neurodegeneration in MSA [12, 14]. Our results in the Olig001-SYN mouse model of MSA show that Tbet deficiency attenuates Olig001-SYN mediated demyelination at 4 (Fig. 3) and 6 months (Fig. 4). More specifically, blocking IFNγ expression or signaling via neutralizing antibody attenuates demyelination (Fig. 6). Under healthy conditions, cytokines and chemokines can affect oligodendroglial differentiation [23, 24, 27]. Our results in Tbet -/- mice indicate that Tbet deficiency did not affect oligodendrocyte cell number and maturation (Additional file 1: Fig. S2E–G), nor did it effect Olig001 transduction (Additional file 1: Fig. S2D). The results from these studies indicate under neuroinflammatory conditions, IFNγ signaling is upstream of demyelination, and by blocking IFNγ expression or activity, demyelination and ultimately neurodegeneration can be prevented (Fig. 4). Based on the data provided in this study, oligodendrocytes are responsive to IFNγ in MSA, however it is unclear if the effect of IFNγ is directly on oliogodendrocytes or mediated through activity of adjacent cells such as microglia or T cells resulting in demyelination. Future studies are needed to understand the mechanisms behind α-syn mediated demyelination and neurodegeneration in MSA.

To model MSA in a mouse model, we used modified AAV model, Olig001-SYN to overexpress α-syn in oligodendrocytes. The Olig001-SYN model recapitulates neuroinflammation, demyelination and neuronal loss in the striatum, all of which are critical parallel pathologies in MSA. Our current and previously published results show that neuroinflammation and demyelination are critical pathologies that occur early in the Olig001-SYN model [35] and persist for the duration of disease pathogenesis (Fig. 4C, Additional file 1: Fig. S4). While the Olig001-SYN model has been useful in understanding the role of neuroinflammation in early disease pathogenesis, additional characterization warranted. While there is significant NeuN+ loss in the striatum of WT mice (Additional file 1: Fig. S3B–C), we did not observe a significant loss of TH+ neurons in the SNpc (Additional file 1: Fig. S3D) at 6 months post transduction. In support, general motor behavior was also assessed at 6-months, and there were no differences in distance traveled and velocity the mice displayed (Additional file 1: Fig. S6). This lack of a motor behavior phenotype could be due to the lack of TH+ neuronal loss in the SNpc at 6-months (Additional file 1: Fig. S3D). Previous studies of Olig001-SYN transduced non-human primates and rats that show there is NeuN+ cell loss within the striatum and TH+ cell loss in the SNpc, however no impaired motor phenotype at 6 months post-injected in non-human primates was observed. The motor behavior phenotype in rats was not investigated [22]. Future studies are warranted to determine if longer time points result in significant TH+ neuron loss in the SNpc and disruption in motor behavior in Olig001-SYN transduced mice.

The Tbet -/- mouse has been useful in understanding how IFNγ drives MSA pathology in the Olig001-SYN model. During this study, additional phenotypes of the Tbet -/- mouse were observed. At baseline, an overall decrease in NeuN+ cells (Fig. 4B) without altered oligodendroglia and myelin (Additional file 1: Fig. S2F and G) were observed in Tbet -/- mice. Interestingly, the Tbet -/- mice also displayed a hyper-active motor phenotype. At a 6-month time point, the Tbet -/- mice had increase velocity and traveled a further distance than the WT mice (Additional file 1: Fig. S6B). However, our findings are not the first to show a behavioral phenotype associated with IFNγ signaling [9]. Further studies are warranted to confirm the specific Tbet-mediated mechanism involved in neurodevelopment and motor behavior.

A limitation of this study was the lack of neutralizing IFNγ long term to assess for neurodegeneration. We assessed that knocking out Tbet attenuates neurodegeneration (Fig. 4), but Tbet deficiency can affect other pathways rendering it was not entirely specific for IFNγ. Utilizing XMG1.2 we were able to neutralize IFNγ, and this led to decreased MHCII expression (Fig. 5B and C), decreased infiltration of monocytes and T cells (Fig. 5B, D, and E), and preserved myelin (Fig. 6). These results suggest IFNγ drives neuroinflammation and demyelination in the Olig001-SYN model. Due to repeated i.p. injections over a 6 month time, assessment of neurodegeneration was not performed in this study, and an IFNγ knockout mouse was not utilized due to severe immune defects [6]. Interestingly, literature has shown neuroinflammation and demyelination occur before neurodegeneration in MSA [8, 12, 17]. Based on that literature, and the attenuated neuroinflammation and demyelination that occurred when IFNγ was neutralized (Figs. 5 and 6), we predict blocking IFNγ signaling will prevent neurodegeneration. A future direction of this project is to directly block IFNγ expression in a cell specific manner and assess for neurodegeneration within the Olig001-SYN model.

Using a novel Thy1.1/IFNγ reporter mouse model, we were able to show that the majority of Olig001-SYN mediated IFNγ production originates from infiltrating CD4+ T cells. This, combined with the observation that Tbet -/- mice show attenuated neuroinflammation, demyelination, and neurodegeneration (Figs. 1, 2, 3 and 4), strongly suggests that IFNγ producing effector T cells potentially drive disease pathogenesis in the Olig001-SYN mouse model of MSA. Although our data suggests that IFNγ producing T cells may be the main driver of MSA pathology in the Olig001-SYN mouse model, they are not the only CD4+ T cell subtype associated with inflammatory or autoimmune disorders [15, 16, 20, 32]. Th17 cells have been shown to induce demyelination and inflammation in MS [15, 16, 32] via production of the proinflammatory cytokine IL-17a [20]. While increases in IL-17a was observed in the Olig001-SYN model in previous studies [35], using a RORγT -/- mouse, we were able to show that this pathogenic subtype does not significantly contribute to the neuroinflammation and demyelination in this model, again supporting the notion that Th17 cells are not key to disease progression (Additional file 1: Fig. S1). Future studies in human post-mortem brain and blood are warranted to validate these results and determine the role of Th1 and Th17 cells in MSA pathogenesis.

In addition to genetic knockout studies, we also showed that neutralizing IFNγ via XMG1.2 treatment was effective in attenuating the neuroinflammation and demyelination in the Olig001-SYN model (Figs. 5 and 6). The neutralization of IFNγ not only highlighted the importance of IFNγ in facilitating demyelination, but it also identifies a potential disease-modifying therapeutic modality in MSA. While the neutralization studies did not reflect a clinical treatment regiment, the results are promising and warrant future investigation for treatment during early disease. Currently, there are no disease modifying treatments for MSA [8, 28], only limited symptomatic treatments. The symptomatic treatments do not extend the lifespan of MSA patients, as they do not halt or slow disease progression. Current clinical trials are designed to target the accumulation of α-syn pathology, or active/passive immunization [17, 28]. Although early results from preclinical models were promising, most trials were terminated in early phases due to failure to meet primary endpoints or the clinical trial sizes were extremely small. While results in animal models suggest targeting neuroinflammation would be promising [19, 34], new clinical trials have targeted the robust neuroinflammatory response observed in MSA. Approaches to suppress activated microglia or astrogliosis failed in phase II due to patients still presenting with neuroinflammation and rapid clinical decline indicating that a peripheral cell type may be driving disease pathogenesis [17, 28]. Intravenous immunoglobin (IVIG) therapy targeting reactive T cells has shown promising phase II results, however, due to the small trial size, this intervention needs further study and development. The current study identifies the IFNγ pathway as a potential disease modifying therapeutic target in a novel mouse model of MSA. Future studies are needed to determine the timing for optimal therapeutic benefit and to determine whether targeting IFNγ attenuates neurodegeneration in the human disease.

In conclusion, the results from these studies show that IFNγ, produced by CD4+ T cells is a key facilitator of neuroinflammation, demyelination, and neurodegeneration as a result of α-syn overexpression in the Olig001-SYN mouse model of MSA. Specifically, genetic knockout or pharmacological approaches targeting IFNγ expression or signaling attenuated CNS microglial activation, and infiltration of pro-inflammatory monocytes and CD4+ T cells. Additionally, targeting IFNγ expression or signaling attenuated Olig001-SYN mediated demyelination, and ultimately neurodegeneration. Using a novel Thy1.1/IFNγ reporter mouse, we determined that IFNγ was expressed primarily by infiltrating CD4+ T cells in response to α-syn overexpression, suggesting Th1 CD4+ T cells are key in facilitating inflammation and disease progression. These results indicate that IFNγ represents a potential future disease-modifying therapeutic target in MSA.

## Supplementary material

Supplementary material is available at *Acta Neuropathologica Communications* online.

### Supplementary Information

**Additional file 1:** **Fig. S1**. Neuroinflammation and demyelination in RORgT -/- mice when α-syn is present **Fig S2**. Neuronal, pSer129+, oligodendrocyte characterization of naïve and Olig001- SYN injected Tbet -/- mice. **Fig S3**. Neurodegeneration characterization of Olig001-SYN in a WT mouse. **Fig S4**. Neuroinflammation 6 months post Olig001-Syn or GFP injection in WT and Tbet - /- mice. **Fig S5**. GCI pathology in WT mice pretreated with XMG1.2. **Fig S6**. Open field data from WT and Tbet -/- 6-months post Olig001-GFP/SYN transduction.

## Abbreviations

α-syn: Alpha-synuclein
CNS: Central nervous system
APC: Antigen presenting cells
GCI: Glial cytoplasmic inclusions
IFNγ: Interferon gamma
IFNγR1: Interferon gamma receptor 1
MHCII: Major histocompatibility complex II
TH: Tyrosine hydroxylase
MSA: Multiple system atrophy
PD: Parkinson’s disease

## References

[1] Arellano G, Ottum PA, Reyes LI, Burgos PI, Naves R (2015). Stage-specific role of interferon-gamma in experimental autoimmune encephalomyelitis and multiple sclerosis. Front Immunol.

[2] Barcia C, Ros CM, Annese V, Gomez A, Ros-Bernal F, Aguado-Yera D, Martinez-Pagan ME, de Pablos V, Fernandez-Villalba E, Herrero MT (2011). IFN-gamma signaling, with the synergistic contribution of TNF-alpha, mediates cell specific microglial and astroglial activation in experimental models of Parkinson's disease. Cell Death Dis.

[3] Bettelli E, Sullivan B, Szabo SJ, Sobel RA, Glimcher LH, Kuchroo VK (2004). Loss of T-bet, but not STAT1, prevents the development of experimental autoimmune encephalomyelitis. J Exp Med.

[4] Cao B, Chen X, Zhang L, Wei Q, Liu H, Feng W, Chen Y, Shang H (2020). Elevated percentage of CD3(+) T-cells and CD4(+)/CD8(+) ratios in multiple system atrophy patients. Front Neurol.

[5] Compta Y, Dias SP, Giraldo DM, Perez-Soriano A, Munoz E, Saura J, Fernandez M, Bravo P, Camara A, Pulido-Salgado M (2019). Cerebrospinal fluid cytokines in multiple system atrophy: a cross-sectional Catalan MSA registry study. Parkinsonism Relat Disord.

[6] Dalton DK, Pitts-Meek S, Keshav S, Figari IS, Bradley A, Stewart TA (1993). Multiple defects of immune cell function in mice with disrupted interferon-gamma genes. Science.

[7] Deczkowska A, Baruch K, Schwartz M (2016). Type I/II interferon balance in the regulation of brain physiology and pathology. Trends Immunol.

[8] Fanciulli A, Wenning GK (2015). Multiple-system atrophy. N Engl J Med.

[9] Filiano AJ, Xu Y, Tustison NJ, Marsh RL, Baker W, Smirnov I, Overall CC, Gadani SP, Turner SD, Weng Z (2016). Unexpected role of interferon-gamma in regulating neuronal connectivity and social behaviour. Nature.

[10] Harms AS, Thome AD, Yan Z, Schonhoff AM, Williams GP, Li X, Liu Y, Qin H, Benveniste EN, Standaert DG (2018). Peripheral monocyte entry is required for alpha-Synuclein induced inflammation and Neurodegeneration in a model of Parkinson disease. Exp Neurol.

[11] Harrington LE, Janowski KM, Oliver JR, Zajac AJ, Weaver CT (2008). Memory CD4 T cells emerge from effector T-cell progenitors. Nature.

[12] Hsiao JT, Tanglay O, Li AA, Strobbe AYG, Kim WS, Halliday GM, Fu Y (2023). Role of oligodendrocyte lineage cells in multiple system atrophy. Cells.

[13] Ivashkiv LB (2018). IFNgamma: signalling, epigenetics and roles in immunity, metabolism, disease and cancer immunotherapy. Nat Rev Immunol.

[14] Jellinger KA (2018). Multiple system atrophy: an oligodendroglioneural synucleinopathy1. J Alzheimers Dis.

[15] Jin M, Gunther R, Akgun K, Hermann A, Ziemssen T (2020). Peripheral proinflammatory Th1/Th17 immune cell shift is linked to disease severity in amyotrophic lateral sclerosis. Sci Rep.

[16] Kamali AN, Noorbakhsh SM, Hamedifar H, Jadidi-Niaragh F, Yazdani R, Bautista JM, Azizi G (2019). A role for Th1-like Th17 cells in the pathogenesis of inflammatory and autoimmune disorders. Mol Immunol.

[17] Lemos M, Wenning GK, Stefanova N (2021). Current experimental disease-modifying therapeutics for multiple system atrophy. J Neural Transm (Vienna).

[18] Levescot A, Cerf-Bensussan N (2022). Regulatory CD8(+) T cells suppress disease. Science.

[19] Lim S, Chun Y, Lee JS, Lee SJ (2016). Neuroinflammation in Synucleinopathies. Brain Pathol.

[20] Liu Z, Qiu AW, Huang Y, Yang Y, Chen JN, Gu TT, Cao BB, Qiu YH, Peng YP (2019). IL-17A exacerbates neuroinflammation and neurodegeneration by activating microglia in rodent models of Parkinson's disease. Brain Behav Immun.

[21] Mandel RJ, Marmion DJ, Kirik D, Chu Y, Heindel C, McCown T, Gray SJ, Kordower JH (2017). Novel oligodendroglial alpha synuclein viral vector models of multiple system atrophy: studies in rodents and nonhuman primates. Acta Neuropathol Commun.

[22] Marmion DJ, Rutkowski AA, Chatterjee D, Hiller BM, Werner MH, Bezard E, Kirik D, McCown T, Gray SJ, Kordower JH (2021). Viral-based rodent and nonhuman primate models of multiple system atrophy: Fidelity to the human disease. Neurobiol Dis.

[23] Meijer M, Agirre E, Kabbe M, van Tuijn CA, Heskol A, Zheng C, Mendanha Falcao A, Bartosovic M, Kirby L, Calini D (2022). Epigenomic priming of immune genes implicates oligodendroglia in multiple sclerosis susceptibility. Neuron.

[24] Pandey S, Shen K, Lee SH, Shen YA, Wang Y, Otero-Garcia M, Kotova N, Vito ST, Laufer BI, Newton DF (2022). Disease-associated oligodendrocyte responses across neurodegenerative diseases. Cell Rep.

[25] Papp MI, Kahn JE, Lantos PL (1989). Glial cytoplasmic inclusions in the CNS of patients with multiple system atrophy (striatonigral degeneration, olivopontocerebellar atrophy and Shy-Drager syndrome). J Neurol Sci.

[26] Powell SK, Khan N, Parker CL, Samulski RJ, Matsushima G, Gray SJ, McCown TJ (2016). Characterization of a novel adeno-associated viral vector with preferential oligodendrocyte tropism. Gene Ther.

[27] Sadick JS, O'Dea MR, Hasel P, Dykstra T, Faustin A, Liddelow SA (2022). Astrocytes and oligodendrocytes undergo subtype-specific transcriptional changes in Alzheimer's disease. Neuron.

[28] Sidoroff V, Bower P, Stefanova N, Fanciulli A, Stankovic I, Poewe W, Seppi K, Wenning GK, Krismer F (2022). Disease-modifying therapies for multiple system atrophy: where are we in 2022?. J Parkinsons Dis.

[29] Sosa RA, Murphey C, Robinson RR, Forsthuber TG (2015). IFN-gamma ameliorates autoimmune encephalomyelitis by limiting myelin lipid peroxidation. Proc Natl Acad Sci USA.

[30] Starhof C, Winge K, Heegaard NHH, Skogstrand K, Friis S, Hejl A (2018). Cerebrospinal fluid pro-inflammatory cytokines differentiate parkinsonian syndromes. J Neuroinflammation.

[31] Tu PH, Galvin JE, Baba M, Giasson B, Tomita T, Leight S, Nakajo S, Iwatsubo T, Trojanowski JQ, Lee VM (1998). Glial cytoplasmic inclusions in white matter oligodendrocytes of multiple system atrophy brains contain insoluble alpha-synuclein. Ann Neurol.

[32] Tuzlak S, Dejean AS, Iannacone M, Quintana FJ, Waisman A, Ginhoux F, Korn T, Becher B (2021). Repositioning TH cell polarization from single cytokines to complex help. Nat Immunol.

[33] Ubhi K, Low P, Masliah E (2011). Multiple system atrophy: a clinical and neuropathological perspective. Trends Neurosci.

[34] Valera E, Spencer B, Fields JA, Trinh I, Adame A, Mante M, Rockenstein E, Desplats P, Masliah E (2017). Combination of alpha-synuclein immunotherapy with anti-inflammatory treatment in a transgenic mouse model of multiple system atrophy. Acta Neuropathol Commun.

[35] Williams GP, Marmion DJ, Schonhoff AM, Jurkuvenaite A, Won WJ, Standaert DG, Kordower JH, Harms AS (2020). T cell infiltration in both human multiple system atrophy and a novel mouse model of the disease. Acta Neuropathol.

[36] Williams GP, Schonhoff AM, Jurkuvenaite A, Gallups NJ, Standaert DG, Harms AS (2021). CD4 T cells mediate brain inflammation and neurodegeneration in a mouse model of Parkinson's disease. Brain.

