# Supplementary material for: IFNγ drives neuroinflammation, demyelination, and neurodegeneration in a mouse model of multiple system atrophy
Source: Acta Neuropathol Commun. 2024 Jan 18;12:11. doi: 10.1186/s40478-023-01710-x (PMC10797897; doi:10.1186/s40478-023-01710-x)
Supplement: Supplementary file 1 — Additional file 1: Fig. S1. Neuroinflammation and demyelination in RORgT -/- mice when α-syn is present Fig S2. Neuronal, pSer129+, oligodendrocyte characterization of naïve and Olig001- SYN injected Tbet -/- mice. Fig S3. Neurodegeneration characterization of Olig001-SYN in a WT mouse. Fig S4. Neuroinflammation 6 months post Olig001-Syn or GFP injection in WT and Tbet - /- mice. Fig S5. GCI pathology in WT mice pretreated with XMG1.2. Fig S6. Open field data from WT and Tbet -/- 6-months post Olig001-GFP/SYN transduction. [file 40478_2023_1710_MOESM1_ESM.docx]

# Supplementary material


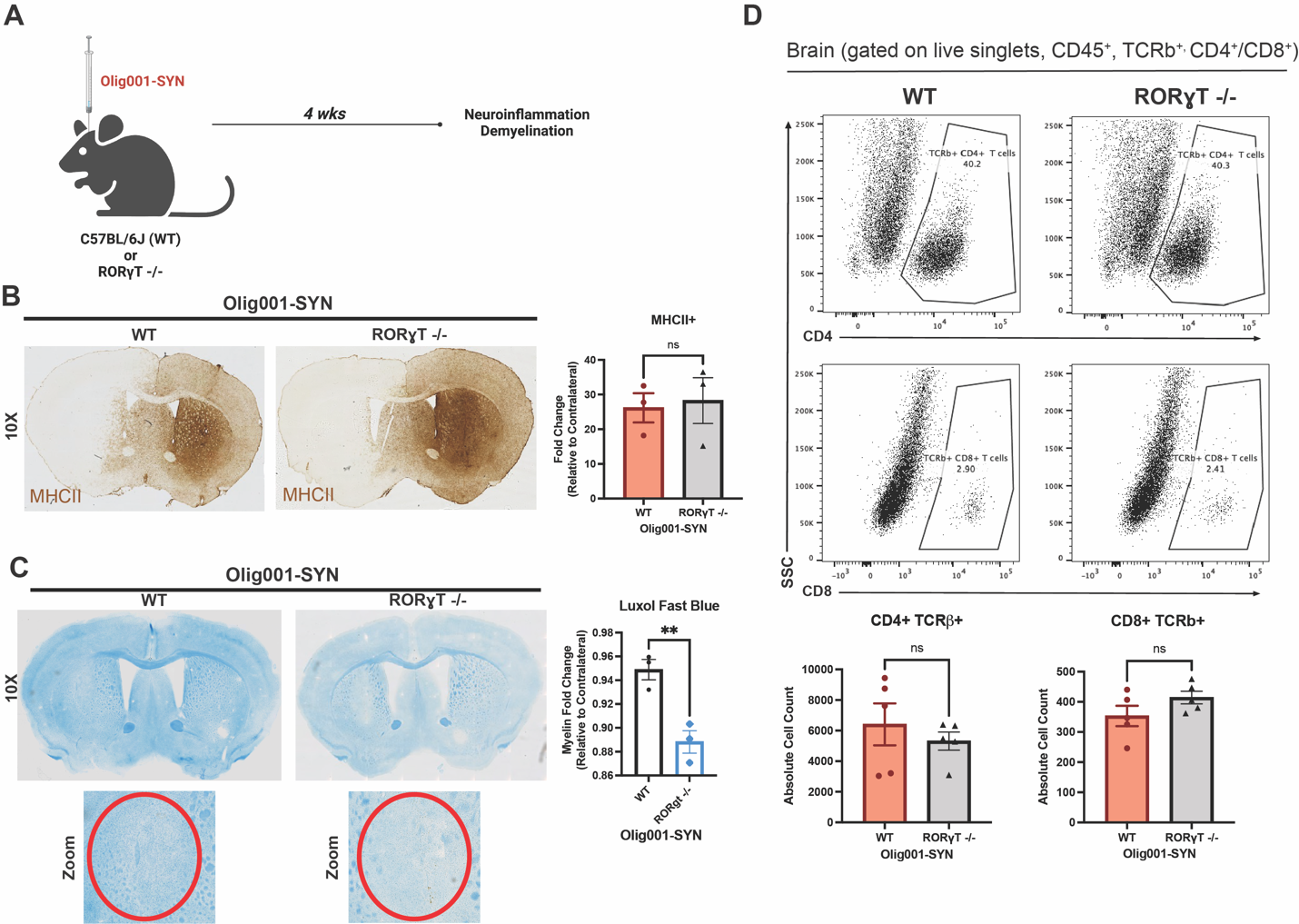


**Supplementary Figure 1: ROR**γT **-/- does not attenuate Olig001-SYN driven neuroinflammation and demyelination.** (**A**) Both male and female RORγT *-/-* mice and WT controls were transduced with Olig001-SYN at 8-12 weeks old. 4 weeks post transduction, tissue was collected and stained with DAB and Luxol Fast Blue to determine demyelination in the striatum and corpus collosum. (**B**) Representative DAB images of WT and RORγT *-/-* mice where MHCII staining is highlighted by staining in the striatum. Quantification between the ispi- and contralateral sides of the striatum in WT and RORγT *-/-* mice were calculated using fold change of staining intensity relative to the contralateral side of the brain. Mean values +/- SEM are plotted, non-parametric Wilcoxon test, ns = no significance. (**C**) Representative images of Luxol Fast Blue staining of WT and RORγT -/- mice. Red circle indicates the area of demyelination. To the right is the quantification of the fold change between the ispi- and contralateral sides. Mean values +/- SEM are plotted, non-parametric Wilcoxon test, **p < 0.01. (**D**) Dot plots representing CD4+ (CD45+, CD11b-, TCRb+, CD4+) and CD8+ (CD45+, CD11b-, TCRb+, CD8+) T cells between WT and RORγT *-/-* mice. Quantification for CD4+ and CD8+ T cell count are graphed below. Mean values +/- SEM are plotted, non-parametric Wilcoxon test. For immunohistochemistry experiments, n=3 per group. For flow cytometry experiments n=5 (two mouse striatum tissues pooled per n) per group. All data points represent one mouse.


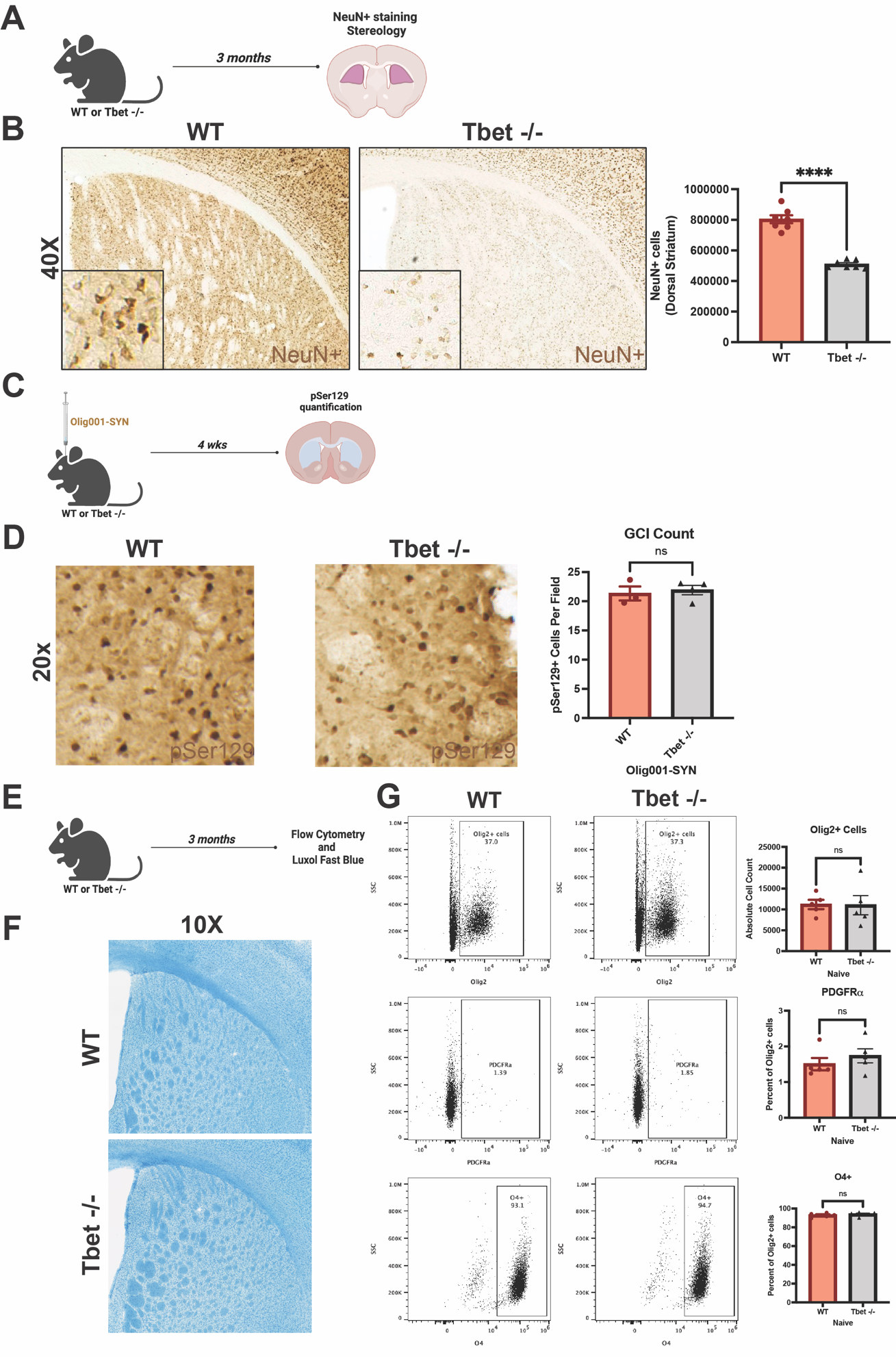


**Supplementary Figure 2: NeuN+, pSer129, and oligodendrocyte characterization of Tbet -/- mice.** (**A**) 3 month old Naïve WT and Tbet -/- were sacrificed and tissue was stained for NeuN+ to assess for the estimated population of neurons in the striatum. (**B**) Representative zoom images that were used for stereology. Quantification of the NeuN+ stereology is to the right of the images. Mean values +/- SEM are plotted, student’s t-test, ****p < 0.0001. (**C**) WT and Tbet -/- at 8 weeks old were unilaterally injected with Olig001-SYN. After 4 weeks, tissue was collected and stained for pSer129. (**D**) Representative images of a counting field used to count the amount of GCI within the striatum of WT and Tbet -/- mice when α-syn is expressed. Mean values +/- SEM are plotted, non-parametric Wilcoxon test, ns= no significance. (**E**) striatum tissue from Naïve WT and Tbet -/- were collected and processed for flow cytometry. (**F**) Representative images of luxol fast blue stain of naïve WT and Tbet -/- mice. (**G**) Absolute cell count of Olig2+ cells (oligodendrocytes). Below are the dot plots of OPCs and early mature oligodendrocytes, and to the left is their respected quantification. Mean values +/- SEM are plotted, non-parametric Wilcoxon test, ns= no significance. For stereology experiments, n=7 per group. For immunohistochemistry experiments, n=4-5 per group. Each data point represents one mouse.

**
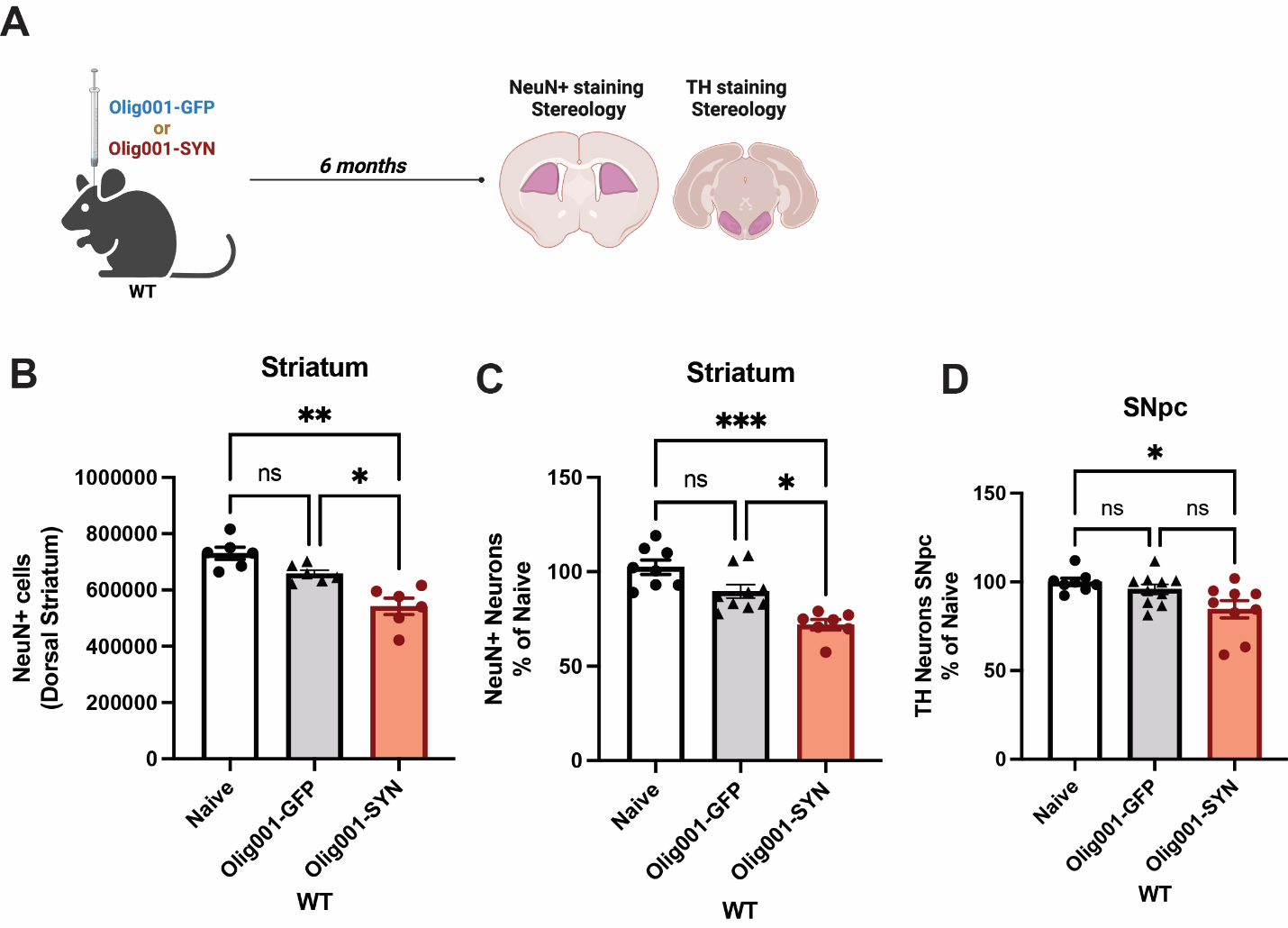
**

**Supplementary Figure 3: Neurodegeneration characterization of Olig001-SYN in a WT mouse.** (**A**) 8-12 weeks old mice were injected with Olig001-GFP or Olig001-SYN. After 6 months, tissue was collected for stereology. The pink contours on the brain cross section define the counting contours used for stereology. This experiment was conducting independently of 6-month study in Figure 4. (**B**) Quantification of NeuN+ stereology in the dorsal lateral striatum. Mean values +/- SEM are plotted, one-way ANOVA with Tukey post hoc for significance, ns= no significance, * p<0.05, ** p<0.01, n=6 per group. (**C**) Quantification of the percent of NeuN+ neurons compared to naïve control within the dorsal striatum. Mean values +/- SEM are plotted, one-way ANOVA with Tukey post hoc for significance ns= no significance, * p<0.05, *** p<0.001, n=8-10 per group. (**D**) Quantification of the percent of TH+ neurons in the substantia nigra para compacta (SNpc) compared to naïve control. Mean values +/- SEM are plotted, one-way ANOVA with Tukey post hoc for significance, ns=no significance, * p<0.05.

**
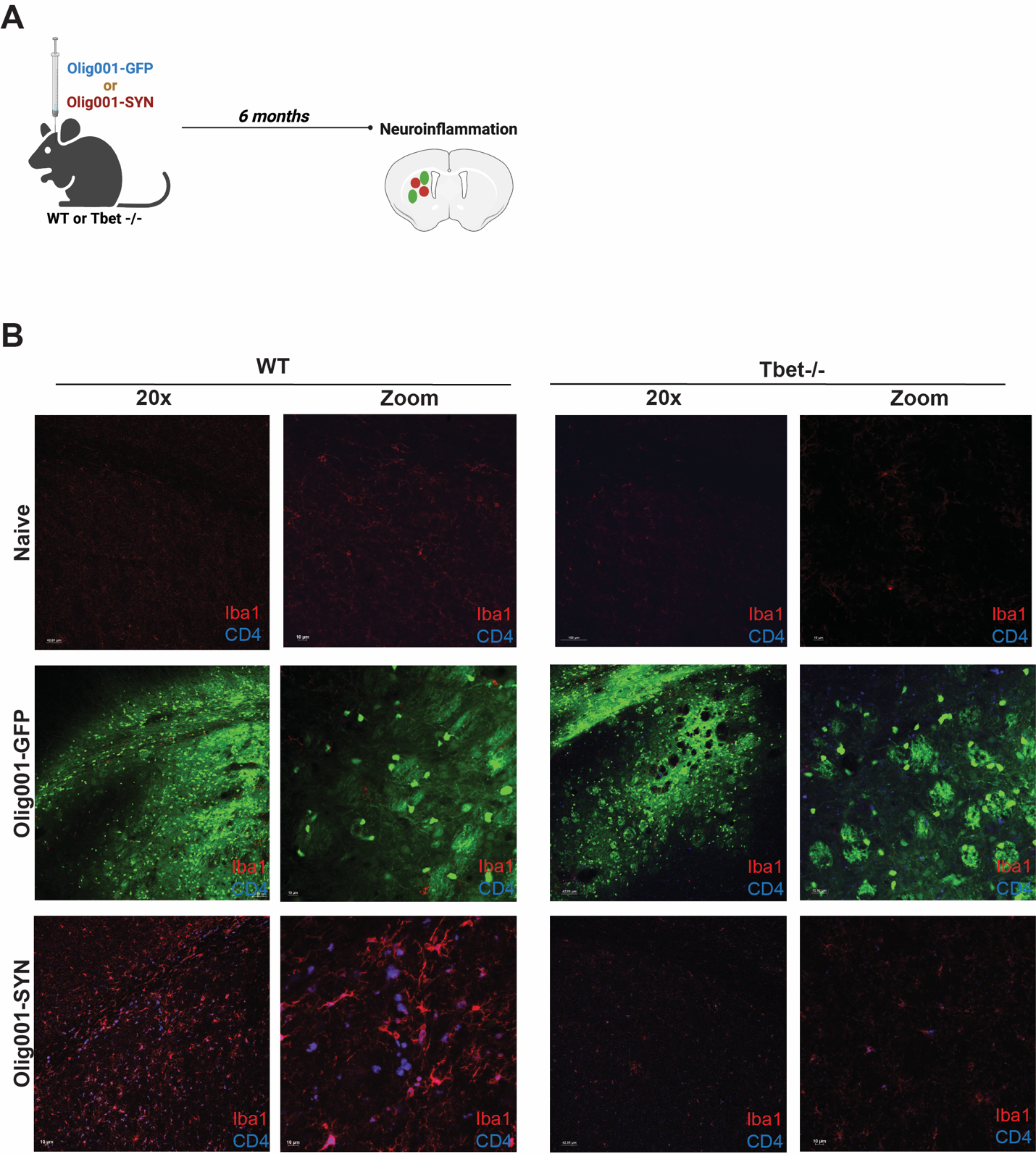
**

**Supplementary Figure 4: Neuroinflammation does not occur in Olig001-SYN injected Tbet -/- mice 6 months post transduction.** (**A**) WT and Tbet -/- mice injected with either Olig001-GFP or SYN were age to 6-months post injection. At 6 months, tissue was collected to assess for neuroinflammation. (**B**) Representative images of tissue stain for Iba1 (red) and CD3 (blue). GFP (green) is expressed in tissue that received Olig001-GFP. For 20x images, the scale bar is set to 50uM and for the zoomed images the scale bar is set to 10 uM.

**
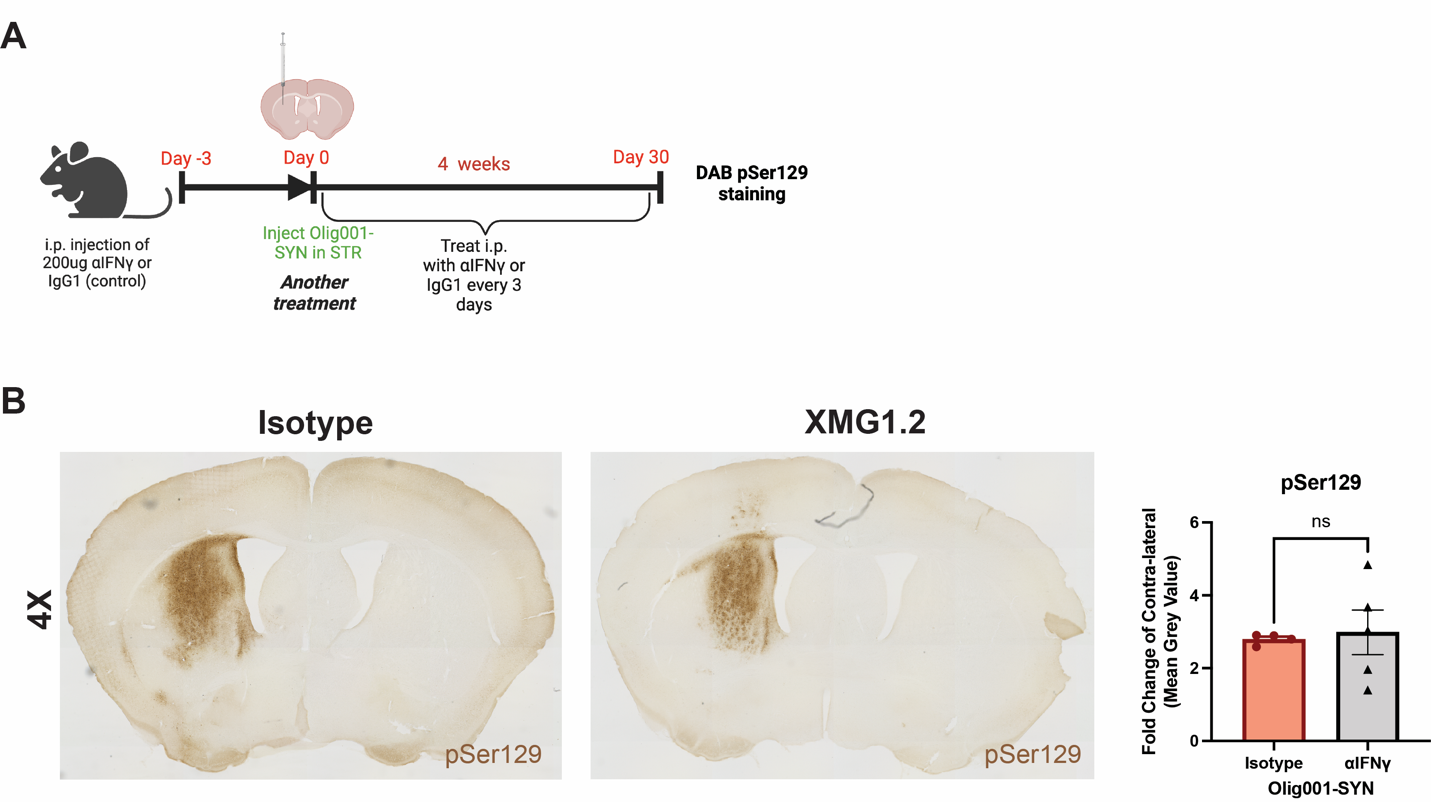
**

**Supplementary Figure 5: GCI pathology does not change upon pre-treatment of XMG1.2.** (**A**) WT mice were pretreated three days before Olig001-SYN was injected into the dorsolateral striatum. When mice were injected with Olig001-SYN, mice received another dose of their respective treatment. Mice received their respective treatment every three days for the course of 30 days. At 30 days, tissue was harvested for IHC. (**B**) Representative DAB images of tissue sections stained for pSer129, and the quantified fold change of ispi- and contra-lateral of pSer129 is on the right. Mean values +/- SEM are plotted, non-parametric Wilcoxon test. For immunohistochemistry experiments, n=4-5 per group.


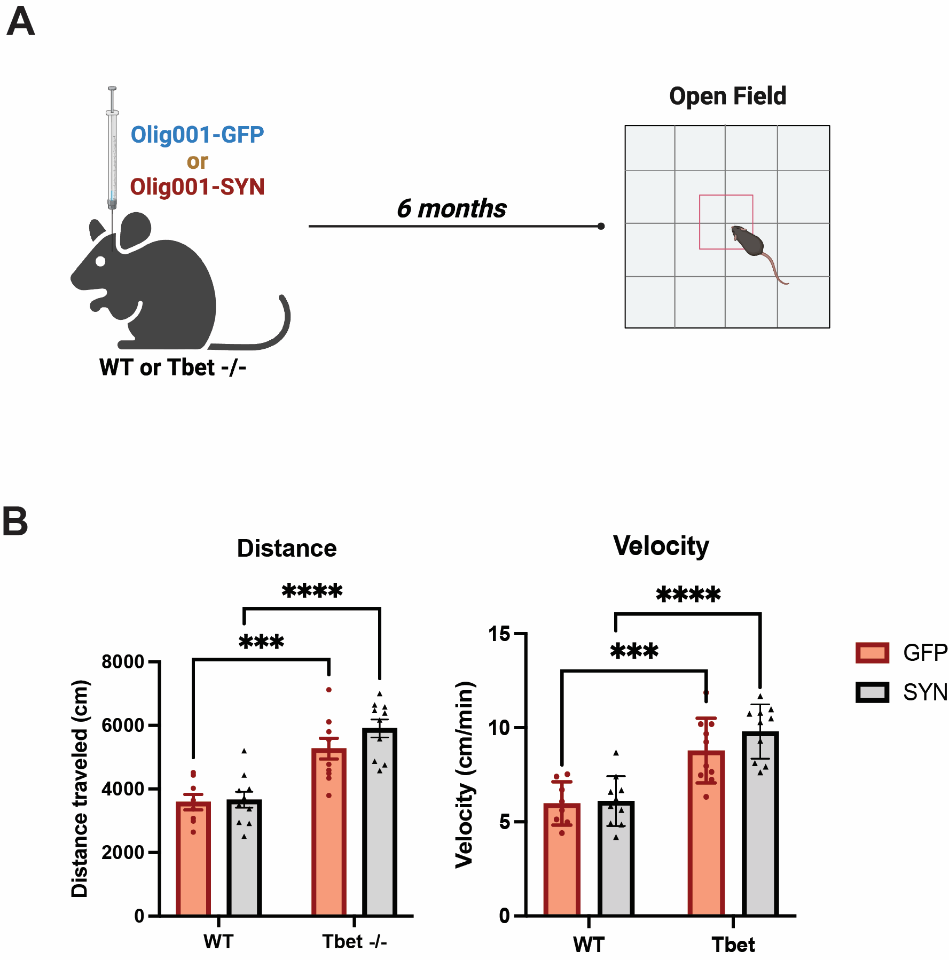


**Supplementary Figure 6: Behavior performed on WT and Tbet -/- injected with either Olig001-GFP or Olig001-SYN.** (**A**) 8-12 week old WT and Tbet -/- mice were bilaterally injected with Olig001-GFP or Olig001-SYN and at 6 months post-inject mice underwent open field. (**B**) For open field, mice were allowed to explore their box for 15 minutes. After 15 minutes mice were returned to their home cage. Distance and velocity were recorded. Mean values +/- SEM are plotted, two-way ANOVA with Tukey post hoc for significance, *** p<0.001, ****p<0.0001. For all behavior task, n=10 mice per group.
